# Variable Suites of Non-effector Genes Are Co-regulated in the Type III Secretion Virulence Regulon across the *Pseudomonas syringae* Phylogeny

**DOI:** 10.1371/journal.ppat.1003807

**Published:** 2014-01-02

**Authors:** Tatiana S. Mucyn, Scott Yourstone, Abigail L. Lind, Surojit Biswas, Marc T. Nishimura, David A. Baltrus, Jason S. Cumbie, Jeff H. Chang, Corbin D. Jones, Jeffery L. Dangl, Sarah R. Grant

**Affiliations:** 1 Department of Biology, University of North Carolina at Chapel Hill, Chapel Hill, North Carolina, United States of America; 2 Program in Bioinformatics and Computational Biology, University of North Carolina at Chapel Hill, Chapel Hill, North Carolina, United States of America; 3 School of Plant Sciences, The University of Arizona, Tucson, Arizona, United States of America; 4 Department of Botany and Plant Pathology, Oregon State University, Corvallis, Oregon, United States of America; 5 Molecular and Cellular Biology Program, Oregon State University, Corvallis, Oregon, United States of America; 6 Center for Genome Research and Biocomputing, Oregon State University, Corvallis, Oregon, United States of America; 7 Curriculum in Genetics and Molecular Biology, University of North Carolina at Chapel Hill, Chapel Hill, North Carolina, United States of America; 8 Lineberger Comprehensive Cancer Center, University of North Carolina at Chapel Hill, Chapel Hill, North Carolina, United States of America; 9 Carolina Center for Genome Sciences, University of North Carolina at Chapel Hill, Chapel Hill, North Carolina, United States of America; 10 Department of Microbiology and Immunology, University of North Carolina at Chapel Hill, Chapel Hill, North Carolina, United States of America; 11 Howard Hughes Medical Institute, University of North Carolina at Chapel Hill, Chapel Hill, North Carolina, United States of America; Massachusetts General Hospital, Harvard Medical School, United States of America

## Abstract

*Pseudomonas syringae* is a phylogenetically diverse species of Gram-negative bacterial plant pathogens responsible for crop diseases around the world. The HrpL sigma factor drives expression of the major *P. syringae* virulence regulon. HrpL controls expression of the genes encoding the structural and functional components of the type III secretion system (T3SS) and the type three secreted effector proteins (T3E) that are collectively essential for virulence. HrpL also regulates expression of an under-explored suite of non-type III effector genes (non-T3E), including toxin production systems and operons not previously associated with virulence. We implemented and refined genome-wide transcriptional analysis methods using cDNA-derived high-throughput sequencing (RNA-seq) data to characterize the HrpL regulon from six isolates of *P. syringae* spanning the diversity of the species. Our transcriptomes, mapped onto both complete and draft genomes, significantly extend earlier studies. We confirmed HrpL-regulation for a majority of previously defined T3E genes in these six strains. We identified two new T3E families from *P. syringae* pv. *oryzae* 1_6, a strain within the relatively underexplored phylogenetic Multi-Locus Sequence Typing (MLST) group IV. The HrpL regulons varied among strains in gene number and content across both their T3E and non-T3E gene suites. Strains within MLST group II consistently express the lowest number of HrpL-regulated genes. We identified events leading to recruitment into, and loss from, the HrpL regulon. These included gene gain and loss, and loss of HrpL regulation caused by group-specific *cis* element mutations in otherwise conserved genes. Novel non-T3E HrpL-regulated genes include an operon that we show is required for full virulence of *P. syringae* pv. *phaseolicola* 1448A on French bean. We highlight the power of integrating genomic, transcriptomic, and phylogenetic information to drive concise functional experimentation and to derive better insight into the evolution of virulence across an evolutionarily diverse pathogen species.

## Author Summary

*Pseudomonas syringae* are environmentally ubiquitous bacteria of wide phylogenetic distribution, which can cause disease on a broad range of plant species. Pathogenicity requires the master regulator HrpL. HrpL controls the activation of virulence factor genes, including those encoding the type III secretion system which facilitates translocation of bacterial proteins into host cells. Here we overlaid transcriptome profiling of genes onto their phylogenetic distribution by characterizing the HrpL regulon across six diverse strains of *P. syringae*. We identified novel putative virulence factors, discovered two novel effector families, and functionally characterized an operon most likely involved in secondary metabolism that we show is required for virulence. We demonstrated that the size and composition of the HrpL regulon varies among strains, and explored how genes are recruited into, or lost from, the virulence regulon. Overall, our work widens the understanding of *P. syringae* pathogenicity and presents an experimental paradigm extensible to other pathogenic bacterial species.

## Introduction

Many Gram-negative bacteria attach to host cells and translocate effector proteins into them via type III secretion systems (T3SS). Such systems are necessary for pathogenesis, are horizontally transferred across species, and are accompanied by dynamically evolving repertoires of type III effector (T3Es) genes [1], [2]. The T3SS is essential for *Pseudomonas syringae* pathogens to thrive in plant tissues. *P. syringae* represents an excellent example of the plasticity of T3E repertoires [3]. Despite a collectively broad host range for the species, individual isolates of *P. syringae* typically display pathogenic potential on a limited set of plants and either elicit immune responses, or simply fail to thrive on other plant species. Strains can be isolated from diseased plants, as epiphytes from healthy plants [4], and from various environmental sources [5].

The *hrp/hrc* group I T3SS is essential for *P. syringae* pathogens to cause disease on plants [1], [6]. The genes that encode the *hrp/hrc* T3SS and accessory proteins are clustered in a conserved pathogenicity island in *P. syringae* [7]. The genes for the associated T3Es can be scattered across the genome, often in association with mobile elements indicative of horizontal transmission [8]–[10]. Each strain's T3E repertoire ranges from 15–30 genes sampled from at least 57 different families and these collectively modify host cell biology to suppress immune response and favor bacterial proliferation and dispersion. However, the action of individual T3E proteins can be recognized by plant host disease resistance proteins, and this triggers immune responses sufficient to limit pathogen growth [11]. The conflicting selective pressures to retain a collection of T3E sufficient to suppress host defenses without triggering effector-specific immune responses [11] drives diversity in the suites of T3Es in plant pathogenic *P. syringae* isolates [3].

Transition from saprophytic to epiphytic or pathogenic lifestyle requires significant transcriptional reprogramming. Expression of genes encoding the *P. syringae* T3SS structural components and the associated T3E suite is controlled by the ECF-type sigma factor HrpL [12]–[14]. The expression of *hrpL* is induced in bacteria that encounter the leaf environment [13]. Subsequently, HrpL binds to promoters carrying a “*hrp*-box” consensus sequence to up-regulate the expression of the corresponding gene(s) [12]–[15].

Previous studies in *P. syringae* identified proteins that are neither T3Es nor structural components of the T3SS (hereafter, non-T3Es), but are HrpL-regulated [3], [16]–[19]. Non-T3Es coordinately regulated with the T3SS and its substrates were also found in other T3SS-expressing plant pathogens such as *Erwinia amylovora* [20], *Ralstonia solanacearum* [21], *Xanthomonas campestris* pv. *vesicatoria* [22], [23] and *Pectobacterium carotovora* [24]. Some HrpL-regulated non-T3E genes affect virulence on host plants in the well-studied strain *P. syringae* pv. *tomato* DC3000 (*Pto*_DC3000_); these include the *corR* regulator of coronatine toxin production [18], [25]. Notably, CorR expression is not HrpL-regulated in other strains, such as *P. syringae* pv. *glycinea* PG4180 [26].

Multi-Locus Sequence Typing (MLST) separates plant pathogenic *P. syringae* into at least 5 distinct phylogenetic groups [3], [27]. The fifth group, represented initially by *P. syrinage pv. maculicola* ES4326, was recently renamed *P. cannabina* pv. *alisalensis* ES4326 [28]. Many *P. syringae* genome sequences are now available, including three closed genomes from isolates representing major pathogen clades [29]–[31], and ∼120 additional draft sequences. Newly sequenced genomes also trace *P. syringae* disease outbreaks across the globe and over time [3], [32]–[39] attesting to the continued importance of the species. Recently, isolation and sequencing of saprophytic and epiphytic strains provided insight into a subgroup from group II that carries a non-canonical T3SS [40]. To date, transcriptome analyses using high throughput short read cDNA sequencing (RNA-seq) have been applied only to *Pto*_DC3000_, providing a well-curated reference gene annotation, but not specifically informing studies of the HrpL regulon [41]–[44].

In this study, we defined the HrpL regulon of six distinct strains of *P. syringae* with complete or draft genomes using RNA-seq coupled with the GENE-counter software package [45]–[47]. We sought primarily to compare the diversity of non-T3E HrpL-regulated genes between strains and secondarily to determine if there were additional type III effectors not found in our DNA-based analyses [3]. We detect non-T3E genes regulated directly or indirectly by HrpL. Those directly regulated by HrpL are distributed throughout the *P. syringae* clades in a mosaic pattern. However, most are either absent or not HrpL-regulated in MLST group II. We demonstrate that a novel cluster of non-T3E genes is required for *P. syringae* pv. *phaseolicola* 1448A virulence. We also identified two novel T3E families from a previously understudied clade. Our study reveals the mechanisms for gene recruitment into, and loss from, the key virulence regulon in *P. syringae*, and provides a roadmap for future functional studies.

## Results

### The HrpL regulons of six phylogenetically diverse *P. syringae* isolates are defined by RNA-seq

We defined the HrpL regulons of *P. syringae* pv. *phaseolicola* strain 1448A (*Pph*_1448A_), *P. syringae* pv. *lachrymans* strain 107 (*Pla*_107_) representing MLST group III; *P. syringae* pv. *syringae* strain B728a (*Psy_B728a_*), *P. syringae* pv. *japonica* strain MAFF 301072 PT (*Pja*) representing MLST group II; *P. syringae* pv. *tomato* strain DC3000 (*Pto*_DC3000_) representing MLST group I and *P. syringae* pv. *oryzae* strain 1_6 (*Por*), belonging to the relatively poorly studied clade, MLST group IV [3], [27]. The native *hrpL* gene from each isolate was cloned downstream of an arabinose-inducible promoter for controlled, high-level expression in the strain of origin. Isogenic strains carrying either the appropriate *hrpL* construct, or an empty vector (EV) as negative control, were grown with arabinose to induce the expression of the cloned *hrpL* gene in a minimal medium [19]. Expression of the native *hrpL* was repressed by addition of peptone to the media [48]. Figure S1 depicts our experimental pipeline and control validation.

We generated Illumina cDNA libraries from two biological replicates of each strain. Because our goal was to compare transcript abundance more than to improve annotation of transcribed genes, we used a simple cDNA method to minimize the RNA processing steps where transcripts could be lost. Therefore, we did not enrich for 5′ ends or distinguish transcript orientation. Transcript abundance was compared between isogenic HrpL and EV samples using GENE-counter [45]. Similar to other RNA-seq analysis methods like EdgeR or DESeq [49], [50], GENE-counter determines differential expression. While EdgeR and DESeq use the standard negative binomial distribution, GENE-counter relies on the negative binomial p distribution which better accounts for the over-dispersion observed in mRNA-seq data [51]–[53]. We bootstrapped the GENE-counter output for each isolate (Materials and Methods) to control for noise introduced by sample normalization. Between 1.6 and 5.6 million unambiguous reads per sample (mapping to only one location in the reference genome) were used for our analyses (Table 1). The sequencing depth ranged from 9.5 to 16.2 times the genome size, with the exception of the *Psy*_B728a_ samples, which we sequenced to higher coverage (Table 1). On average 93.5% of the total number of annotated coding genes in a genome were covered by at least one read in at least one sample (Table 1). Bootstrapped-GENE-counter analysis established a median read count for every sample, a median *q*-value and a *B*-value, for every gene covered by at least one read in one biological replicate (Table S1). Genes not covered by any unambiguous reads are not represented in our GENE-counter output. The *B*-value represents the percentage of bootstraps in which a particular gene was called differentially expressed.

**Table 1:** Summary of Illumina RNA-seq data.

	*Pph*_1448A_	*Pla_1_*_07__ Draft	*Psy*_B728a_	*Pja*_ Draft	*Por*_ Draft	*Pto*_DC3000_	*Pto*_DC3000__ Draft
Technology	GAII	GAII	Hi-seq	GAII	GAII	GAII	GAII
read length (nt)	36	36	50	36	36	36	36
Average # of reads used for analysis after bootstrapping	2,376,423	1,613,243	5,675,482	2,506,746	2,652,065	1,971,190	1,836,589
Average # of nt used for analysis after bootstrapping	85,551,228	58,076,775	283,774,100	90,242,856	95,474,340	70,962,840	66,117,204
Genome size (bp)	6,112,448	6,030,058	6,093,698	6,932,599	5,886,178	6,538,260	6,924,419
Sequencing depth*	14.0	9.6	46.6	13.0	16.2	10.9	9.5
# coding genes in genome	5,172	6,744	5,089	9,534	6,329	5,619	5,618
# coding genes covered by reads	5,047	6,362	5,061	7,414	5,951	5,317	5,573

* The sequencing depth was defined as the number of nt used for the analysis divided by the size of the genome.

We further considered only genes with *B*-values≥50%. Like all “significance thresholds” the *B*-value cut-off is somewhat subjective. We selected a *B*-value of 50% to apply to all genomes because this threshold captured 95% of the known HrpL-regulated genes identified in our control genome, *Pto*_DC3000_, with a median *q*-value greater than 0.05. We identified between 59 to 192 genes differentially expressed across the strains (Table S1). For all strains, the large majority of the differentially expressed genes were up-regulated (between 53 and 180 genes, Table 2). These genes mainly encode T3SS components and known T3Es. Surprisingly, we identified few HrpL-down-regulated genes (Table S2): ranging from none in *Pph*_1448A_ to 45 in *Pla*_107_. Genes called down-regulated in our analysis had relatively low *q*-values, reflecting low differences in read coverage between HrpL and EV samples. Lan et al. 2006 and Ferreira et al. 2006 identified down-regulated genes in their microarray studies for *Pto*_DC3000_. However, almost no overlap was found between the list of down-regulated genes from previous studies and ours, indicating that the down-regulated genes identified are most likely neither biologically, nor statistically robust, and thus unlikely to be biologically relevant. In contrast, there was stronger overlap between our HrpL-induced genes and those shared between these earlier studies (see below). Down-regulated genes were therefore not further analyzed. Finally, we manually inspected and curated all genes with *B*-values greater than or equal to 50% to define the HrpL regulon for each strain (Table 2, Table S3; Table S4; Materials and Methods).

**Table 2:** Characterization of the HrpL-regulon across *Pseudomonas syringae* strains.

	*Pph*_1448A_	*Pla_1_*_07__ Draft	*Psy*_B728a_	*Pja*_ Draft	*Por*_ Draft	*Pto*_DC3000_	*Pto*_DC3000__ Draft
Genes up by RNA-seq raw	88	167	63	78	232	133	131/124^e^
Genes up B-value≥50%	76	108	53	69	180	115	ND
Genes up after curation^a^	71	90	51	46	114	110	ND
% of known HrpL-genes found by RNA-seq^b^	79%	NA	NA	NA	NA	91%	87%
# of genes expected to be HrpL-regulated by RNA-seq^c^	58	62	45	38	82	96	ND
# of putative novel virulence genes identified by RNA-seq	13	28	6	8	32	14	ND
# of missing genes^d^	8	NA	NA	NA	NA	5	ND
Genes down B-value≥50%	0	45	5	12	12	38	ND

a , See Materials and Methods.

b , The number of known HrpL-regulated genes for *Pto*_DC3000_ was determined using data collected by [16], [17], [19], and defined as the number of genes found up-regulated in at least two studies. For *Pph*_1448A_ the number of known HrpL-regulated genes was determined according to data generated by [18], [19]; and defined as the number of genes found up-regulated in either study. See Table S5 for details.

c , Genes expected to be HrpL-regulated were defined as genes being part of an operon known to be HrpL-regulated, genes found HrpL-dependent in other strains, or genes involved in coronatine synthesis. See Table S6 for details.

d , Missing genes were defined as “ known HrpL-regulated” not found differentially expressed in our analysis.

e , represent the number of genes found upregulated after genes split up between contigs were removed. NA, not applicable. ND, not determined.

### Analysis of RNA-seq data is reproducible

**Figure 1:**
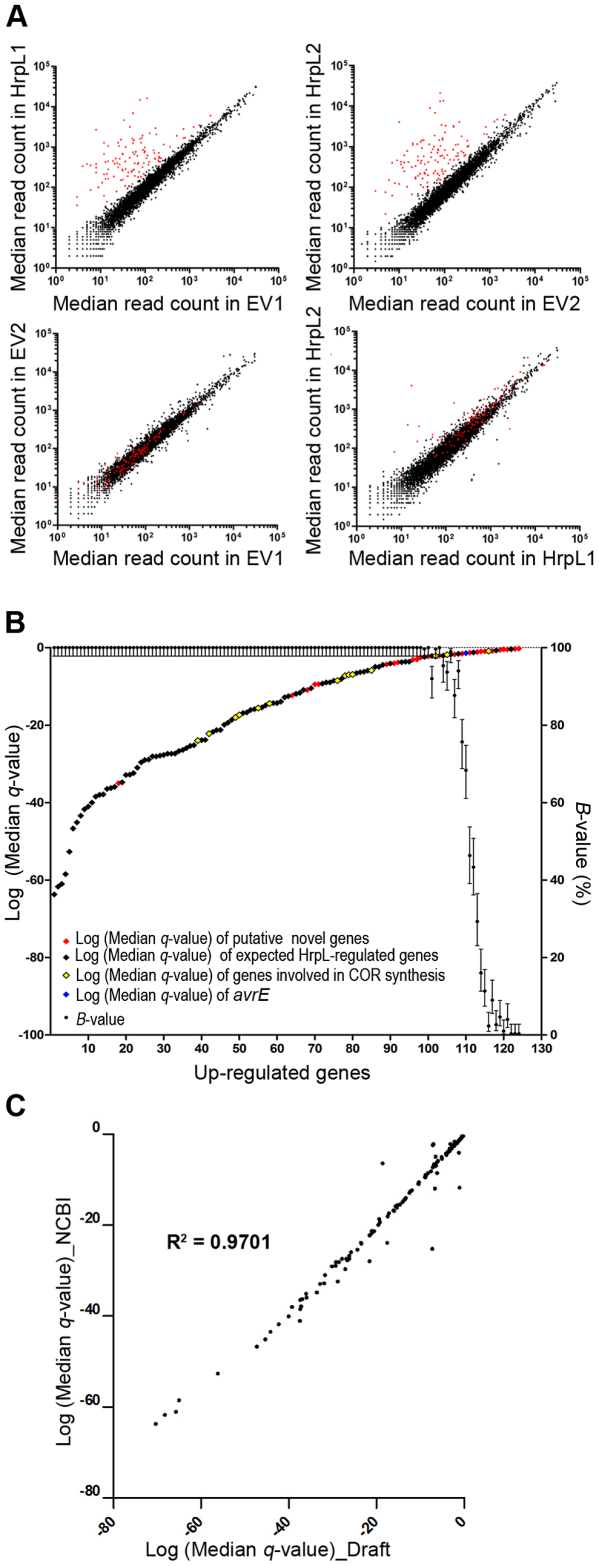
Validation of our RNA-seq method. (**A**) Read coverage of *Pto*_DC3000_ genes within and between biological replicates displays high reproducibility. A graphical logarithmic representation of the median read counts of all genes covered by unique reads after bootstrapping of the data is presented. Top panels: comparison of *Pto*_DC3000_(pBAD::*hrpL*) and *Pto*_DC3000_(pBAD::EV) samples within (left) the first (HrpL1 or EV1), or the second (HrpL2 or EV2) (right) biological replicate. Bottom panels: comparison of *Pto*_DC3000_(pBAD::EV) samples (left), and *Pto*_DC3000_(pBAD::*hrpL*) samples (right) across two biological replicates. Red dots represent the logarithmic median read count of genes found to be significantly up-regulated in the presence of HrpL by GENE-counter with a *B*-value≥50%. (**B**) Graphical representation of GENE-counter data for *Pto*_DC3000_ bootstrapped 300 times. Genes identified as differentially up-regulated were plotted according to their Log (Median *q*-value). Data points were color-coded for putatively novel genes (genes not previously described as HrpL-regulated, in red), genes previously described as HrpL-regulated, *hrp* complex genes, type III effector genes (in black), and genes involved in coronatine synthesis (in yellow). *avrE* is marked in blue. (**C**) Draft and complete (NCBI) *Pto*_DC3000_ reference genomes yield highly similar RNA-seq results. Every gene found up-regulated with a *B*-value≥50 using both NCBI and draft genome as reference genomes was plotted according to its Log (median *q*-value).

To evaluate the reproducibility of our method, we compared the read coverage within and between biological samples for all *Pto*_DC3000_ genes covered by at least one read in our normalized GENE-counter data set. Biologically replicated samples had highly correlated results (R^2^ = 0.93 between EV replicates 1 and 2; R^2^ = 0.96 between conditional expression replicates HrpL1 and HrpL2, Figure 1A, lower panels). Comparing HrpL and EV replicates from two biological replicates, the majority of the data points correlate and cluster around the trend line (Figure 1A, upper panels). The outlier data points in red represent genes defined as differentially up-regulated by GENE-counter and having a *B*-value≥50%. We plotted the log of the median *q*-value of each *Pto*_DC3000_ gene defined to be differentially up-regulated (before manual curation) and their corresponding *B*-values ranked from smallest to largest (Figure 1B). As expected, genes with highly significant *q*-scores also have high *B*-values. Several genes not previously reported to be HrpL-regulated (marked in red) had more significant *q*-value scores (3.8E-02) than *avrE* (marked in blue), a well-characterized conserved HrpL-regulated type III effector [54].

We analyzed the same *Pto*_DC3000_ RNA-seq data set using either the complete *Pto*_DC3000_ genome sequence [30] or a draft *Pto*_DC3000_ genome sequence [36] as references. The draft genome sequence covers 85% of genes at over 90% of their length [36]. Using either the complete or the draft genome as a reference resulted in similar sequencing depths (Table 1). Using the draft genome as a reference, GENE-counter identified 124 HrpL-upregulated genes out of the 133 found using the complete *Pto*_DC3000_ genome (Table 2). Most of the genes that were not identified as differentially expressed using the draft genome were missing from the draft genome (data not shown). The high correlation between the Log(median *q*-value) of genes in the two data sets (Figure 1C) indicates that our method will effectively identify the majority of genes of the HrpL regulon from *P. syringae* isolates for which only a high quality draft genome is available.

### RNA-seq successfully captures the HrpL regulon for *Pto*_DC3000_ and *Pph*_1448A_

To further validate our pipeline to define HrpL–regulated genes, we compared our manually curated list of 110 *Pto*_DC3000_ HrpL-regulated genes (Table 2) to HrpL-regulated genes identified by three previous studies: one promoter probe study using an arabinose-inducible *hrpL* gene and two custom microarray analyses which compared expression between wild type and *hrpL* deletion mutant strains [16], [17], [19]. These studies produced largely overlapping, but not identical, lists of putatively HrpL-regulated genes (Table S5). Our *Pto*_DC3000_ HrpL-regulated gene set included 57 out of the 66 genes previously identified as HrpL-regulated in at least two of the previous studies (Table S5), even though our induction and analysis methods differed from these studies. 96 of the 110 genes we identified were also found to be HrpL-regulated in at least one of the previous studies [16], [17], [19] or were downstream genes in HrpL-regulated operons (Table 2). Overall, we found 91% of the previously identified HrpL-regulated genes in *Pto*_DC3000_. Our analysis also identified 14 novel HrpL-regulated genes (Table 2); six out of eight tested were confirmed to be HrpL-regulated using qRT-PCR (Table 3, see below).

**Table 3:** Real time RT-PCR analyses predominantly confirm RNA-seq data.

Genes tested	Annotation	Median Q-value (Genecounter)	Putative *hrp*-box	pBAD system* #	Fold induction in pBAD system	Native system* ∧	% expression in Δ*hrpL* vs. WT
*PSPTO_4332*	hypothetical protein	4.66E-13	−	+	15.3	+	3.8
*PSPTO_2130*	LuxR family DNA-binding response regulator	3.45E-10	+	+	5.2	+	19.0
*PSPTO_3086*	transcriptional regulator	3.75E-10	−	+	24.0	+	4.1
*PSPTO_2129*	sensory box histidine kinase/response regulator	4.86E-05	−	+	3.4	+	21.3
*PSPTO_2208*	htpG heat shock protein 90	9.82E-05	−	+	2.7	+	53.6
*PSPTO_0871*	macrolide efflux protein	1.51E-04	+	+	2.8	ND	ND
*PSPTO_1843*	aspartate kinase	1.48E-03	−	−	0.9	ND	ND
*PSPTO_4716*	hypothetical protein	1.89E-02	−	−	1.5	ND	ND
*PSPPH_A0112*	phosphoglycerate mutase family protein	3.11E-11	−	+	4.8	+	20.5
*PSPPH_A0110*	hypothetical protein	3.94E-10	−	ND	ND	+	53.6
*PSPPH_A0109*	sulfotransferase, putative	7.96E-08	−	+	4.3	+	51.6
*PSPPH_1906*	LuxR family DNA-binding response regulator	5.51E-07	+	+	4.9	+	4.1
*PSPPH_0762*	hypothetical protein	4.45E-03	−	+	4.4	+	9.9
*PSPPH_A0106*	hypothetical protein	7.49E-03	−	ND	ND	+	46.4
*PSPPH_A0108*	adenosylmethionine-8-amino-7-oxononanoate aminotransferase	1.27E-02	−	ND	ND	+	36.7
*Psyr_0737*	putative transmembrane protein	1.00E-46	+	+	23.3	+	8.0
*Psyr_0027*	hypothetical protein	1.64E-14	−	+	4.3	−	84.5
*PORcurated_00518*	hypothetical protein	2.49E-54	+	+	84.3	+	2.0
*PORcurated_04644*	Methyltransferase small domain	1.16E-37	+	+	48.4	+	3.5
*PORcurated_03530*	hypothetical protein	3.03E-30	+	+	19.5	+	34.2
*PORcurated_04640*	hypothetical protein	1.72E-27	+	+	19.8	+	42.3
*PORcurated_04022*	Alkylated DNA repair protein	4.24E-24	−	+/−	1.7	ND	ND
*PORcurated_04648*	hypothetical protein	2.35E-14	−	+	4.1	+	41.7
*PORcurated_04371*	hypothetical protein	3.39E-24	+	+	5.2	+	30.2
*PORcurated_04025*	hypothetical protein	1.99E-11	−	+	3.4	ND	ND
*PORcurated_04024*	Domain of unknown function (DUF1883)	1.06E-04	−	−	0.6	ND	ND

* + found up-regulated by qRT-PCR; − no up-regulation. ND, not determined. See Figure S2 and S3 for detailed qRT-PCR results.

# expression compared between *Ps* (pBAD::EV) strain and *Ps* (pBAD::*hrpL*) strain grown in media containing arabinose.

∧ expression compared between a wild type strain and an isogenic clean *hrpL* deletion mutant grown in MM media.

Notably, four of the nine missing genes were not present in our laboratory strain, which has lost part of the *Pto*_DC3000_ plasmid A. One gene, *shcA* (*PSPTO_5353*) was found differentially expressed in our analysis but had a *B*-value less than 50%. Further, GENE-counter discards RNA-seq reads that map non-uniquely to more than one location in the genome, and HrpL-regulated duplicated genes account for three missing *Pto*_DC3000_ genes: T3E genes *hopAM1-1*(*PSPTO_1022*) and *hopQ1-2* (*PSPTO_4732*), and the non-T3E gene *plcA2* (*PSPTO_B0005*) (Table S5). Finally, *hopK1* (*PSPTO_0044*), was covered by RNA-seq reads but the differences in expression in HrpL and EV treatments were not statistically significant (Table S1, S5).

Two previous studies focused on the identification of HrpL-regulated genes in *Pph*_1448A_ [18], [19] and identified 43 HrpL-regulated genes comparing expression between wild type and *hrpL* mutants. We identified 35 (∼80%). Four of the missing eight genes were covered by reads but not found significantly differentially expressed, *hopAK1* (*PSPPH_1424*), a gene encoding a MarR transcriptional regulator (*PSPPH_1519*), *avrRps4* (*PSPPH_A0087*), and *hopAS1* (*PSPPH_4736*). Those four genes had a median read coverage ranging from 100 to 1000, indicating that the absence of differential expression in our analysis is not due to weak or undetectable levels of expression. One, *PSPPH_2294* is a pseudogene. *PSPPH_1525* encoding a putative effector related to *Ralstonia* Hpx30 [55], *PSPPH_A0009* and *A00075* encoding truncated *hopW1* are duplicated and had very low to no read coverage (Table S5). Our GENE-counter analysis pipeline results are consistent with previous transcriptional studies, reinforcing the validity of our methods. Additionally, we identified robustly HrpL-induced genes that were not previously identified.

### Quantitative RT-PCR analyses predominantly confirm our RNA-seq data

We identified between six and 32 genes previously not known to be HrpL-regulated in each strain with corresponding *q*-values ranging from E-02 to E-54 (Table 2, Table S3). Some of these are shared across strains. We could not identify a consensus upstream *hrp*-box in the promoters of several, and suggest that these could be indirectly activated by HrpL. We performed qRT-PCR using samples derived from strains expressing HrpL in the pBAD system and confirmed 19 of 23 tested (Figure S2). Additionally, we confirmed HrpL-dependent expression of 19 genes out of 20 tested, by comparing wild type expression with expression in a *hrpL* deletion mutant in *hrpL*-inducing minimal medium (Table 3, Figure S3). We observed a high correlation between RNA-seq data and either qRT-PCR profiling method, especially for genes with a q value>E-03 (Table 3, Figures S2, S3). In sum, we identified the majority of previously identified HrpL-regulated genes in two well-studied strains and we confirmed wild type HrpL regulation for nearly all of the newly identified members of this key virulence regulon.

### RNA-seq identifies new T3E genes

**Figure 2:**
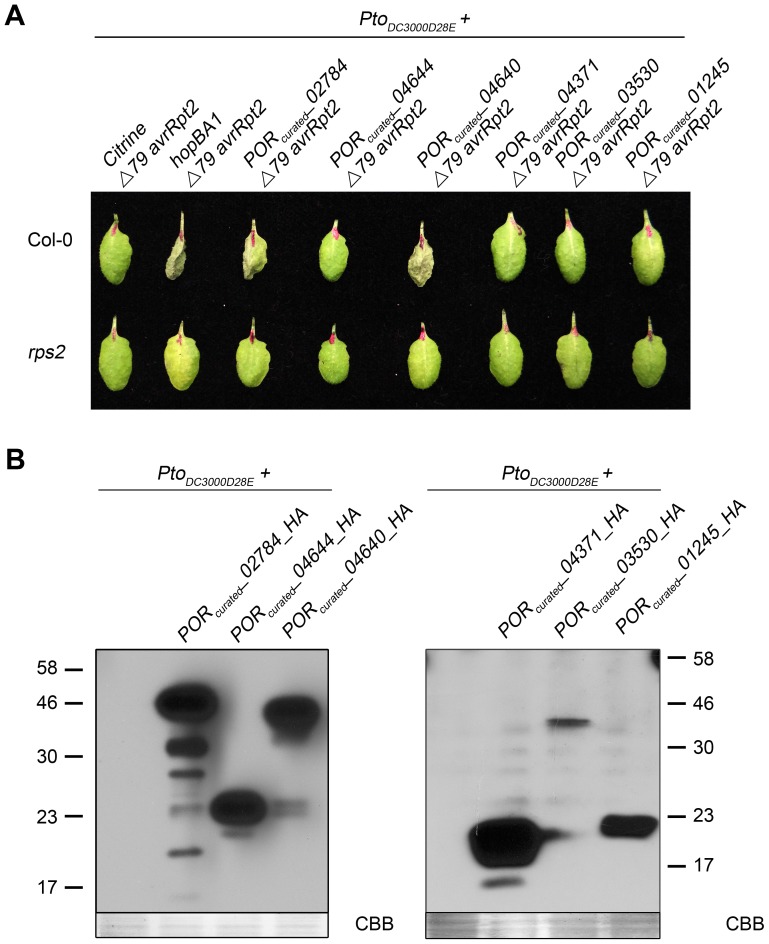
Identification of a novel type III effector. (**A**) Δ79*avrRpt2* translocation assay. Four week old Col-0 and Col-0 *rps2–101c* (*rps2*) plants were hand inoculated with *Pto*_DC300028E_ without plasmid or *Pto*_DC300028E_ carrying either citrine (negative control), *hopBA1* and its upstream region (positive control), IMG/ER *Por_cuarted__02784, 04644, 04640, 03530, 02145, and 04371* sequence and upstream sequence into Δ79*avrRpt2* -fusion vector pJC532. Plants were scored and pictures were taken 24 h after inoculation for hypersensitive response (HR). (**B**) Western blotting analysis. *Pto*_DC300028E_ without plasmid (negative control), *Pto*_DC300028E_ carrying IMG/ER *Por_curated__02784, 04644, 04640, 03530, 02145, and 04371* sequence and upstream region into HA-fusion vector pJC531 were grown on MM media for 5 hours. Culture aliquots were subjected to western blot using anti-HA antibody. CBB, Coomassie brilliant blue.

Most of the known T3E and candidate T3E genes in our tested strains and those previously defined by similarity and/or functional criteria were included in the HrpL regulons we defined in our RNA-seq analyses (Figure S4). Most of strains used in this study had previously been screened for novel type III effector genes by functional translocation assays with the exception of *Por* and *Pja* [3], [19]. Therefore, we searched the *Por* and *Pja* HrpL regulons for potential novel effector genes based on the criteria of having an identifiable upstream *hrp*-box sequence and no homology to previously identified T3E families. We chose six *Por* genes (*Por_curated__02784, 04644, 04640, 03530, 02145, and 04371*) to investigate as potentially encoding novel T3Es. *Pja* also carries a gene homologous to *Por_curated__04644*; but only the *Por* allele was tested. All six putative T3E were tested for their ability to be translocated via a native T3SS using an established assay [56] (Materials and Methods) from *Pto*_DC3000D28E_, an “effector-less” *Pto*_DC3000_ strain [57]. Only *Pto*_DC3000D28E_ carrying *Por*_curated_ *_02784-Δ79avrRpt2* or *Por*_curated_ *_04640-Δ79avrRpt2* triggered a Hypersensitive Response (HR) in Col-0 (Figure 2A). We verified that HA-tagged versions of all six T3E candidates were expressed in *Pto*_DC3000D28E_ indicating that lack of HR in our translocation assay was unlikely due to a lack of protein accumulation (Figure 2B). No HR was observed in the *rps2* mutant, indicating that the response was *avrRpt2*-specific and not the result of toxicity. These two new *P. syringae* effectors will henceforth be referred to as HopBH1*_Por_* and HopBI1*_Por_* according to proposed T3E naming guidelines [58].

None of the 19 *P. syringae* strains for which we previously performed comparative genomic analysis encode either *hopBH1* or *hopBI1* [3]. However, each can be found in *P. syringae* strains isolated from various sources ranging from non-symptomatic plants to snow [33], [35], [40], [59]–[62] (Figure S5). Amino acid sequence alignments suggest that HopBH1 is a bi-modular effector exhibiting sequence conservation within its C-terminal domain and sequence diversity toward its N-terminal half (Figure S6). In the non-pathogenic strain *Psy*_642_, the putative HopBH1 protein appears to have been disrupted by a frameshift mutation, leading to two putative open reading frames designated as ORF29-30 [40]. Phylogenetic analysis of strains carrying either *hopBH1* and/or *hopBI1* indicates that both effector genes occur with a mosaic distribution across the *P. syringae*, but are absent from the phylogenetic group III [3], [27] (Figure S5). Neither HopBH1 nor HopBI1 contain known protein folds, nor do they display sequence or structural homology to proteins of known function.

### The HrpL regulons are diverse across isolates

**Figure 3:**
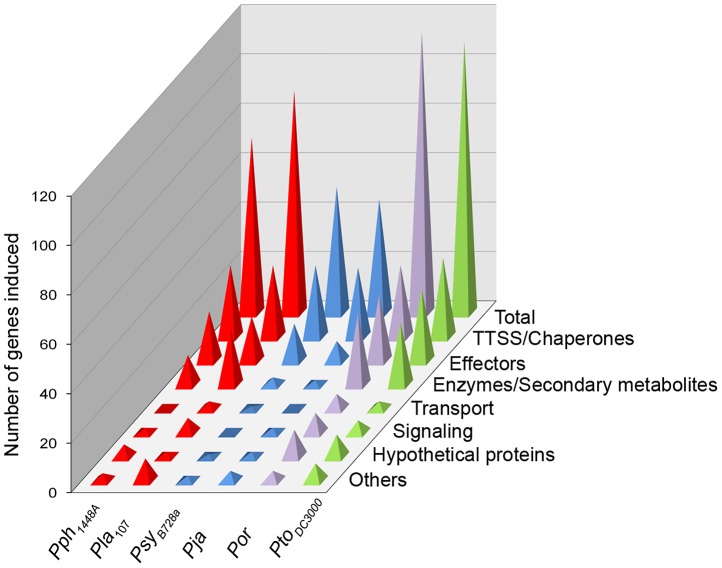
HrpL regulons across phylogenetically distinct strains vary in size and composition. Each gene of every HrpL regulon (after manual curation) was categorized according to its putative function based on annotation, or annotation of its best hit after BLAST against the completely sequenced strains. For details on which category every gene was assigned refer to Table S7.

The composition of the HrpL regulon across strains was surveyed by functional classification based on protein annotation and sequence homology determined by BLASTP (Table S6). As summarized in Figure 3 and Table S7, *Pto*_DC3000_ and *Por* possess the largest and most diverse HrpL regulons among the sampled strains, while the Group II strains *Pja* and *Psy*_B728a_ have the smallest. We are confident that the less complex HrpL regulons are not a sampling artifact, because the data collected from *Pja* has a transcriptome depth similar to the other strains, and the *Psy*_B728a_ HrpL regulon was sampled at relatively high depth compared to our other transcriptomes. We conclude that HrpL regulons vary in size and composition across the *P. syringae* phylogeny.

### Recruitment of genes into and out of the HrpL regulon

**Figure 4:**
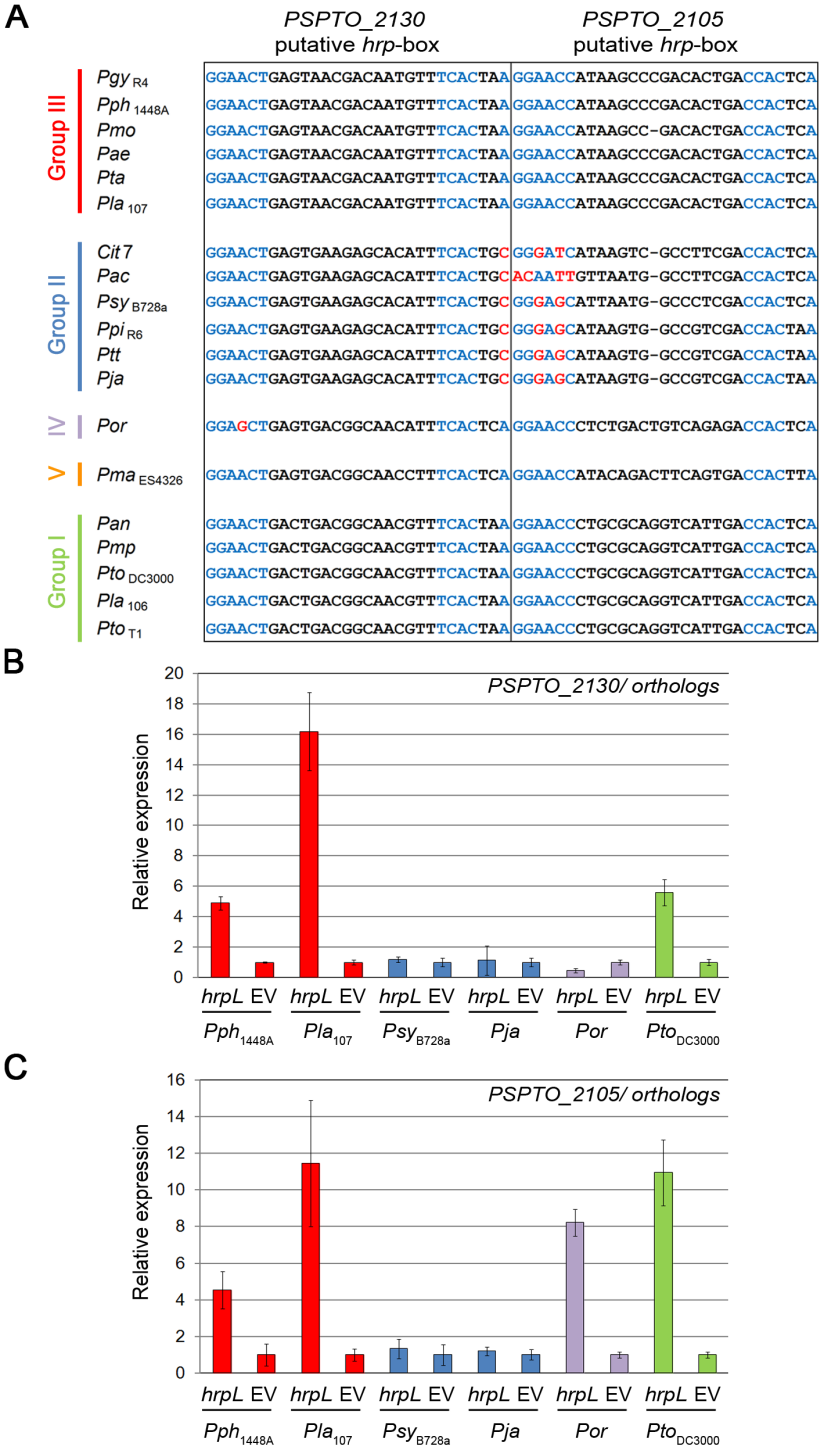
*hrp*-box mutations associated with differential HrpL-dependent up-regulation of *PSPTO_2105, and 2130* orthologs. (**A**) *PSPTO_2105* and *PSPTO_2130 hrp*-box sequence variation across *P. syringae strains* as presented in [3]. In blue, canonical *hrp*-box nucleotides. In red, nucleotides of divergent *hrp*-box sequences. (**B**) HrpL-dependent expression of *PSPTO_2130* orthologs across *P. syringae* strains. qRT-PCR analysis was performed on RNA samples derived from isogenic strains expressing, or lacking, an arabinose-inducible *hrpL* gene. Expression was normalized to *gap-1*. Relative expression: each EV sample was set to 1 and HrpL samples normalized to the corresponding EV samples. Error bars represent SD. (**C**) HrpL-dependent *expression of PSPTO_2105 orthologs across P. syringae strains*, as above. Each experiment was repeated twice.

We observed variable HrpL-dependent expression for several highly conserved non-T3E genes present in all six strains (Table S6). We identified polymorphisms in the *hrp*-box sequences from two of these (Figure 4A). In the first case, new HrpL-regulated genes we identified, *PSPTO_2130, PSPPH_1906* and *Lac107_00061530*, are orthologs that encode a DNA-binding response regulator. HrpL-dependent induction was confirmed by qRT-PCR (Table 3, Figure 4B). Orthologous genes are also present in *Pja*, *Psy*_B728a_, *Por* (*Pjap_00016990*, *Psyr_1940*, and *Por_curated__00527*, respectively) but were not identified as differentially expressed (Table S1). *PSPTO_2130* and all of its orthologs have conserved *hrp*-box motifs. However, the promoters of the orthologs from *Por* and all other group II strains contain single nucleotide polymorphisms (in red, Figure 4A) in the consensus *hrp*-box sequence. Our RNA-seq data suggested that expression of these polymorphic alleles was not HrpL-dependent, a finding confirmed by qRT-PCR performed with both of our HrpL-regulation experimental tests (Figure 4B, Figure S7A).

*PSPTO_2130* and its orthologs are part of a putative operon composed of four genes, *PSPTO_2128-2131* (Figure S8A). Unusually, the *hrp*-box sequences were located within the first ORF of the putative operons of *PSPTO_2130* and its orthologs. We monitored HrpL-dependent expression using qRT-PCR of all genes from *PSPTO_2131* to *2128* from three strains (Figure S8B, C, D). In none of these strains was the first gene of the operon, containing the putative *hrp*-box, differentially expressed. By contrast, HrpL-dependent expression was observed for genes downstream of the predicted *hrp*-box, including coding sequences, *PSPTO_2130* and *PSPPH_1906*, in all but the group II reference strain *Psy*_B728a_ (Figure S8). Deletion mutants in *Pto*_DC300_ and *Pph*_1448A_ of *PSPTO_2130* and *PSPPH_1906* did not display any growth defect on Arabidopsis accession Col-0 or French bean cultivar Tendergreen (susceptible to *Pto*_DC3000_ and *Pph*_1448A_, respectively) (data not shown). Thus, the role of *PSPTO_2130* and its orthologs in virulence remains unclear.

In the second case, *PSPTO_2105* and its orthologs, which encode a putative ApbE-family protein, are highly conserved across *P. syringae* and are HrpL-regulated in *Pph*_1448A_, *Pla*_107_, *Pto_DC3000_* and *Por* but not in the group II strains *Psy*_B728a_ or *Pja* (Table S5, S6). qRT-PCR (Figure 4C, Figure S7B) support our RNA-seq data. *PSPTO_2105* is required for full virulence of *Pto*_DC3000_ on Arabidopsis [18]. We also observed significantly reduced virulence when we tested two independent deletion mutants of the *Pph*_1448A_ ortholog *PSPPH_1855* for growth on the native host, French beans (Figure S9). Every group II strain analyzed has variations in the otherwise well conserved *hrp*-box sequence in at least two positions (Figure 4A). Collectively, these data demonstrate that promoter erosion within the *hrp*-box is a mechanism to remove genes from the virulence regulon.

### HrpL regulons of isolates from phylogenetic group II contain fewer non-T3E genes

**Figure 5:**
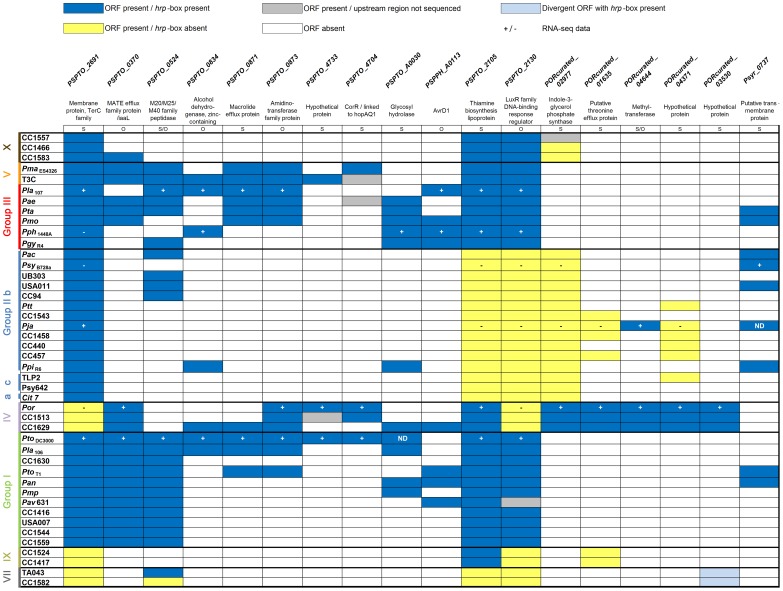
Strains of phylogenetic group II have the fewest non-effector genes in their HrpL regulons. “Non-effector” genes are listed across the top; *Pseudomonas* genomes, color-coded by phylogenetic group, on the left, according to Figure S5 (*Pseudomonas* UB246 was not included, because it belongs to a divergent pseudomonad lineage). Only the first gene of an operon is represented. Dark blue box indicates presence of full-length ORFs (with at least 80% nucleotide identity and 40% coverage), by similarity search as well as the presence of a *hrp*-box in the 500 bp upstream region. Grey boxes indicate that the corresponding gene was present, but the presence of putative *hrp*-boxes could not be determined, due to incomplete sequencing of the upstream region. Yellow boxes indicate that genes are present but either no *hrp*-boxes were detected in the upstream region or *hrp*-boxes are presumably not functional (because divergent from the *hrp*-box for which HrpL-dependent expression was confirmed). White boxes indicate absence of genes based on homology searches. The light blue boxes indicate the presence of a divergent ORF with upstream *hrp*-box sequence present. S, indicates that the gene is present as a single gene. O, indicates that the gene is present in an operon. + or − indicate that the gene was differentially expressed or not in our RNA-seq data. ND indicates not determined: *PSPTO_A00030* is absent from our *Pto*_DC3000_ laboratory strain, and a homologous sequence of *Psyr_0737* is present in *Pja* genome but was not annotated as an ORF.

Both *Psy*_B728a_ and *Pja* appear to have relatively small HrpL regulons; both belong to the MLST group II. To address whether this was a general feature of group II strains, and to address the distribution of the genes that we identified experimentally across the phylogeny, we extended our investigation of non-T3E HrpL regulon diversity to BLAST homology searches of 44 sequenced *Pseudomonas* spp. strains [3], [35], [63]. Our non-T3E gene search set included genes likely to be directly HrpL-regulated, derived from either previous studies [19], [64] or this study. From our study, these included genes we experimentally confirmed for HrpL-dependent expression, genes that encoded proteins found not to be translocated, or genes unlikely to encode a translocated product by annotation. We removed T3SS genes and known T3Es (Figure 5). Most of the directly HrpL-regulated non-T3E genes we identified are absent from group II strains, but distributed across strains from groups I and III. Some are present in the previously described group IV and V, as well as the novel MLST groups VII, IX, X (Berge et al., personal communication, Figure S5) for which we had limited sampling. Further, the promoters of group II homologs of *Por*_curated__*02977, 01635* are divergent, and lack canonical *hrp*-boxes (data not shown). Thus, not only do group II strains possess lower numbers of known T3E genes on average than the other phylogroups, group II strains also possess fewer non-T3E genes in their HrpL regulon suggesting a potential shift in virulence mechanisms of this clade [3].

### Recruitment of a novel gene cluster into an *avrD*-containing virulence operon in *Pph*_1448A_

**Figure 6:**
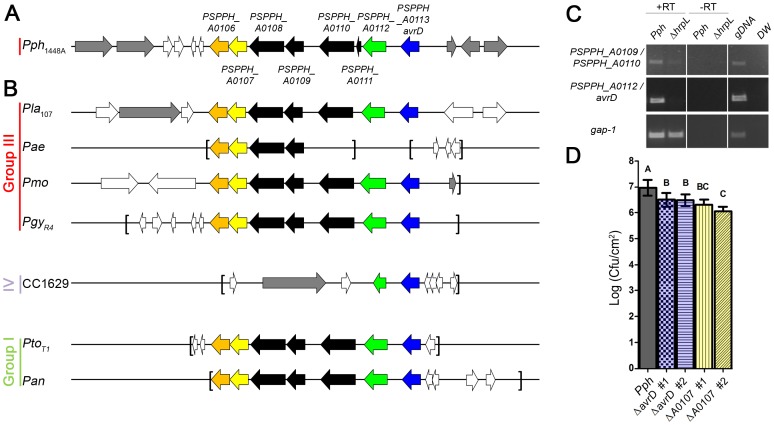
A novel HrpL-regulated virulence operon linked to *avrD* in *Pph*_1448A_ identified by RNA-seq analysis. (**A**) Color coded genomic context of *avrD* and downstream genes from *PSPPH_A0106* to *A0112*. Grey arrows represent transposases. White arrows represent additional ORFs that are not necessary related. (**B**) Graphical representation of *avrD* operon according to IMG/ER conserved neighborhood region search with the 45 *Pseudomonas* strains presented in Figure 5 and S5. Grey, white, black, and colored arrows as in A. Brackets represent breaks and end of contigs or scaffolds. Not to scale. (**C**) PCR was performed using primers spanning intragenic regions between *PSPPH_A0109* and *A0110* (top panels), or *PSPPH_A0112* and *avrD* (middle panels) on cDNA prepared from *Pph*_1448A_ (*Pph*) or an isogenic *Pph*_1448A_Δ*hrpL* mutant (Δ*hrpL*) grown in MM media. Total RNA was subject to reverse transcriptase (+RT), or without reverse transcriptase (−RT, as negative control). gDNA, DW indicate respectively that genomic DNA or distilled water were used as template for positive and negative controls of amplification. Equal loading was controlled by monitoring *gap-1* amplification across samples (bottom panels). (**D**) Two week old bean cv. Tendergreen beans were dip inoculated with wild type *Pph*_1448A_ *(Pph)*, two independent clean deletion *avrD* mutants (Δa*vrD* #1, Δa*vrD* #2), and two independent clean deletion *PSPPH_A0107* mutants (Δ*PSPPH_A0107* #1, Δ*PSPPH_A0107* #2) at OD_600_ = 0.001. Bacterial growth of each strain was determined after 3.5 dpi. Letters represent significant differences with *P*<0.05 according to Tukey's highly significant difference and error bars display standard deviation. This experiment was repeated at least twice.

Both *Pph*_1448A_ and *Pla*_107_ contain *avrD*, a gene required for synthesis of syringolides, small molecules sufficient for HR on soybean cultivars expressing the *Rpg4* disease resistance gene [65]–[67]. *avrD* is a non-T3E gene, as defined above (Figure 5), and its expression in *E. coli* is sufficient for production of syringolides [65]. RNA-seq analysis identified a series of orthologous, HrpL-regulated genes directly downstream of *avrD* in both *Pph*_1448A_ and *Pla*_107_ (Table S3, S6). In *Pph*_1448A_, those genes are arranged in two clusters composed of *PSPPH_A0112-A0110 and PSPPH_A0109-A0106*, which are flanked by transposable elements (Figure 6A). While most of these genes seem to encode hypothetical proteins, PSPPH*_A0112, A0109, A0108, A0107* encode putative enzymes: a phosphoglycerate mutase, a sulfotransferase, an amino transferase, and an oxidoreductase respectively. We confirmed the HrpL-dependent expression of *PSPPH_A0112*, *A0110*, *A0109*, and *A0107* (Table 3, Figure S2, and S3). This operon is typically found as a presence/absence polymorphism; when present, it is almost always downstream from *avrD* (Figure 6B). *PSPPH_A0111* corresponds to a 99 bp sequence present in *Pla*_107_, *P. syringae* pv. *mori* (*Pmo*), *P. syringae* pv. *glycinea* R4 (*Pgy*_R4_), *P. syringae* pv. *tomato* T1 (*Pto*_T1_), and *P. syringae* pv. *actinidiae* (*Pan*) but not annotated as an ORF, thus it is not represented in the graphical representation of the conserved neighborhood region (Figure 6B). In *P. syringae* CC1629, this putative operon appears to have been disrupted by insertion of a transposable element. In *P. syringae* pv *aesculi* 0893_23 (*Pae*) this locus is not entirely sequenced. To determine whether *avrD* is part of an operon with *PSPPH_A0112-A0106*, we used RT-PCR to confirm that the intragenic regions between *avrD/PSPPH_A0112* and between *PSPPH_A0110/PSPPH_A0109* were transcribed in wild type *Pph*_1448A_ but either very weakly or not at all in the Δ*hrpL* mutant (Figure 6C).

We generated two independent deletion mutants for *avrD* and *PSPPH_A0107* (Δ*avrD* #1 and 2, Δ*PSPPH_A0107* # 1 and 2, respectively) and tested their growth on French bean cv. Tendergreen (Figure 6D). All mutants displayed reduced growth compared to wild type *Pph*_1448A_ (*Pph*), indicating that both *avrD* and *PSPPH_A0107* are required for full virulence on cv. Tendergreen. We confirmed that the HrpL-dependent expression of several downstream genes was not disrupted by mutations (Figure S10). However, *PSPPH_A0112, A0107* and *A0106* were consistently slightly up-regulated in *avrD* mutants compared to the wild type. The intact remaining *hrp*-box is closer to *PSPPH_A0112-A0106* in the *avrD* mutants, which could account for increased transcript levels. Additionally, these data could explain why the *avrD* mutants displayed a reduced growth defect compared to the Δ*PSPPH_A0107* mutants (Figure 6D). We speculate that these non-T3E genes are involved in the synthesis of a secondary metabolite(s) required for virulence of *Pph*_1448A_.

## Discussion

*P. syringae* is a broadly distributed and agronomically important pathogen of many plant species. Full virulence for many strains within this species requires expression of genes induced by the sigma factor HrpL, but the HrpL regulon has only been systematically surveyed using microarrays in *Pto*_DC3000_ [16], [17] and to a limited extent by promoter probe studies in a few strains [3], [19]. Using RNA-seq, we successfully defined HrpL regulons across six phylogenetically diverse strains. We benchmarked our data set with previous transcriptional studies of two reference genomes [16]–[19] (Table 2) and with qRT-PCR analysis (Table 3, Figure S2, Figure S3). Our approach allowed us to efficiently define the HrpL regulon of multiple strains, even those for which only draft genome sequence is available. We found a plethora of non-T3E genes in these regulons and experimentally verified both newly identified T3Es and non-T3E virulence factors. Additionally, we identified a variety of mechanisms that could drive recruitment into and loss from, the main virulence regulon of *P. syringae*.

### Identification of two novel HrpL-regulated type III effectors in *Por*

We identified HopBH1*_Por_* and HopBI1*_Por_*, defining two novel effector families. Both have a mosaic phylogenetic distribution across *P. syringae* [35], [40], [63] (and an unpublished strain, TLP2, JGI taxon ID: 2507262033). Both are present in CC1513 and CC1629, two other strains belonging to the MLST group IV. They appear to be absent from sequenced MLST group III strains. HopBH1 has a bi-modular structure. The ∼170 amino-acid N-terminus is divergent compared to the relatively well conserved ∼250 amino acid C-terminal domain across HopBH1 alleles (Figure S6). The HopBH1 C-terminal domain is 50% identical to a protein from *P. fluorescens* SS101 which lacks a putative *hrp*-box or a T3SS secretion competent N-terminal sequence [68], suggesting that it may have been recruited as an effector by N-terminal assortment [69]. Several putative proteins present in *Pantoea, Serratia, Burkholderia* species, as well as *Myxobacteria*, display ∼50% identity with the HopBH1 C-terminal domain. Remarkably, about 150 amino acids of the HopBH1 C-terminal domain also shares 40% identity with part of the ∼1000 amino acid long *P. savastanoi* pv. *savastanoi* NCPPB3335 HrpK. Notably, this *hrpK* gene (*PSA3335_2516*) is from a rhizobia-like type III secretion and is different from the *hrpK(Pto)* (*PSA3335_1389*) of canonical T3SS conserved in plant pathogenic *P. syringae* [70]. HopBI appears to be confined to *Pseudomonas*. Neither HopBH1, nor HopBI1 display similarity to known-effectors. Their virulence functions remain to be determined.

### HrpL regulons include diverse non-T3E genes some of which are known virulence factors

Although analysis of type III virulence systems focuses mainly on the characterization and function of T3SS and T3E proteins, several non-T3E genes are co-regulated with the T3SS. They encode hypothetical proteins, transporters, or enzymes likely involved in secondary metabolism (Figure 5). In contrast to T3E genes, for which functional redundancy is predominant and generation of multiple effector mutants is often required to affect virulence [54], [57], [71], [72], several single knockout mutants of non-T3E HrpL-regulated genes in *Pto*_DC3000_ and *Pph*_1448A_ displayed reduced virulence on Arabidopsis and beans [18], [73]. In general, little is known about the non-T3E genes in HrpL regulons, but homology provides reasonable scenarios for several that we identified, and we functionally validated others (below).

Among our collection of diverse HrpL-regulated, non-T3E genes, none are present in the HrpL regulon of all six strains tested, and nearly all are distributed in a mosaic pattern among the genomes of available strains (Figure 5).

*PSPTO_0370* and orthologs encode a MATE efflux transporter present in an operon with *iaaL* which is involved in auxin conjugation to IAA-Lys [74]. *Por_curated_*_02977 encodes a putative indole-3-glycerol phosphate synthase. Both potentially alter auxin signaling and could interfere with the balance between immune response and growth and development [75].

Several other putative transporters were identified as HrpL-regulated. *PSPTO_2691* encodes a putative membrane protein TerC; *PSPTO_0871* a putative macrolide efflux protein; *Por_curated__01635* a putative threonine efflux protein; and *PSPTO_0838* a putative major facilitator family transporter. Co-regulation of putative transporters with the T3SS suggests that promotion of nutrient acquisition, export of secondary metabolites, or detoxification of plant-encoded antimicrobials are important features of the virulence regulon.

*PSPTO_0834*, encoding a putative alcohol dehydrogenase, is the first gene of a putative operon comprising five genes (up to *PSPTO_0838*). This operon includes genes of unknown function, genes encoding a putative bifunctional deaminase-reductase enzyme and a transporter. The function of this operon remains unknown but at least *PSPTO_0834* is required for full virulence of *Pto*_DC3000_ on Arabidopsis [18].

The *PSPTO_0873-0875* putative operon is widely distributed across *Pseudomonas* and *Erwinia* species and also present in *Pantoea stewartii* pv. *stewartii* DC283. In *Erwinia* and *P. stewartii*, this operon is physically linked to the T3SS and is HrpL-regulated. *PSPTO_0873* is a putative amidinotransferase that makes ornithine and homo-arginine from arginine and lysine. Ornithine or homo-arginine may be then incorporated into a tri- or di-peptide natural product generated by the rest of this operon. Most interestingly, *hsvC, hsvB, hsvA* from *Erwinia amylovora*, corresponding to *PSPTO_0873-0875*, are required for full virulence on apple shoots [76].

*PSPTO_2105* and orthologs encode a protein similar to ApbE from *Salmonella typhimurium* involved in thiamine synthesis. ApbE was identified through the analysis of several mutants defective in thiamine biosynthesis, and was implicated in iron-sulfur cluster biosynthesis/repair, as well as FAD binding [77]–[79] suggesting a role during oxidative stress [78]. *PSPTO_2105* is required for full virulence of *Pto*_DC3000_ on Arabidopsis [18]. We extend this finding by showing that the *PSPPH_1855* ortholog of *PSPTO_2105* is required for full virulence of *Pph*_1448A_ on French bean (Figure S9).

*PSPTO_2130* and orthologs encode LuxR family DNA-binding response regulators that may be involved in regulation of regulons downstream of HrpL. Our deletion mutants of this gene in *Pto*_DC3000_ and *Pph*_1448A_, or of the entire *Pto*_DC3000_ operon, did not alter growth on Arabidopsis or French bean cv. Tendergreen, respectively (data not shown), undermining the probability of a necessary function during plant colonization in our experimental conditions. However this operon is conserved across *Pseudomonas*, and *PFLU_2937*, the ortholog of *PSPTO_2129* from *P. fluorescence* SBW25, was identified as a plant-induced gene [80]. It therefore remains plausible that this operon is involved in plant association.

*Por_curated__04644* appears to encode a putative RNA N-methyltransferase, while the hypothetical protein Por_curated__03530 has homology to FliB which, in *Salmonella*, is responsible for methylation of flagellin [81]. We speculate that both may be involved in modification of conserved molecules known to induce host defense responses [82]–[84].

*avrD* is widely distributed across bacteria and is involved in the synthesis of syringolides [85]. Syringolides are elicitors of cell death in soybean expressing the *Rpg4* disease resistance gene [86], [87]. The putative function of *avrD* is discussed below.

### The group II strains have smaller HrpL regulons

One of our most striking comparative observations is the relatively small size and diversity of the HrpL regulons of the phylogenetic group II strains *Psy_B728a_* and *Pja*. We observed that most of the non-T3E genes known to be HrpL-regulated in other strains are not present, or lack HrpL-regulation in group II strains, underpinning the conclusion that the limited regulon observed for *Psy*_B728a_ and *Pja* can most likely be generalized to all group II strains (Figure 5). They also contain fewer T3Es than the other clades [3]. The group II strains carry genes for phytotoxins not shared by other *P. syringae* groups. Expression of these phytotoxins is not regulated by HrpL, and could compensate for missing T3E functions, making a smaller T3E repertoire sufficient to suppress plant defenses [3].

### Modes of recruitment of non-T3E genes into, and out of, the HrpL regulon

Turnover within the HrpL regulon is known to be influenced by gene gain and loss, mediated by association of genes within the regulon with mobile elements and horizontal gene transfer (data not shown, Figure 6 A, B). However, we also observed that all the group II strains analyzed here have polymorphisms in the *hrp*-box sequence that correlated with the loss of HrpL-dependent regulation of *PSPTO_2105* and orthologs (likely encoding AbpE). Several different polymorphisms within the *hrp*-box were observed, suggesting independent mutational events (Figure 4). Additionally, the group II strain orthologs of *PSPTO_2130* (LuxR family), carry nucleotide polymorphisms in the consensus *hrp*-box, and are not HrpL-regulated (Figure 4). Orthologous genes from *Por* also display nucleotide variation in this *hrp*-box, also leading to loss of HrpL-regulation. The substitution patterns of these alterations suggest multiple, independent losses of HrpL-regulation. *PSPTO_2130* and its orthologs are part of an operon where the consensus *hrp*-box is embedded within the first ORF in this operon (Figure S8) and is thus likely to be constrained by the genetic code. Interestingly, *PSPTO_2130* and its orthologs have variation in the second half of the *hrp* box where CCAC is replaced by TCAC. This *hrp*-box motif, while uncommon, is also found in *PSPTO_0370*, *PORcurated_01251* (*hopAO1_Por_*), and *Pjap_00002060* (*hopC1_Pja_*), each of which we defined as HrpL-regulated.

The promoter erosion we observe could be driven by negative host selection pressure, or weak selection for maintenance of HrpL regulon membership combined with subsequent drift. Similarly, reversion of at least the SNPs could quickly recruit genes back into the HrpL regulon. Because the ORFs have not accumulated stop mutations, these promoter mutations are either relatively recent or there is active maintenance of the ORF sequence, perhaps for expression under different conditions.

Horizontal transfer or other types of recombination could explain how 5′ regions diverge and how these regions and associated genes are recruited in to the HrpL regulon. *Por_curated__02977*, *01635, and 04371*, encode an indole-3-glycerol phosphate synthase, a putative threonine efflux transporter and a hypothetical protein, respectively, that are HrpL-regulated. Similar genes are present in *Pja* and *Psy*_B728a_ but are not HrpL-regulated (Figure 5). Putative *hrp*-boxes can be identified in all three *Por* genes, but not for the corresponding genes in *Pja* and *Psy*_B728a_. These genes are not syntenic (data not shown). They display high similarity in their coding sequence (data not shown); however their corresponding 5′ upstream regions are highly divergent. This could be the result of horizontal transfer, though there is no obvious footprint of mobile element DNA, or independent recombination events.

Lastly, loss of transcription termination regulation could lead to read-through transcription, and thus provide a mechanism for recruitment of non-T3E genes into the HrpL regulon. This mechanism was first highlighted by the recruitment into the HrpL regulon of the *corR* gene which was recombined downstream of the *hrp*-box associated *hopAQ1* gene, in *Pto*_DC3000_ [25]. We observed that several genes found differentially expressed in our analysis were located downstream of HrpL-regulated T3E genes (Table S3) and could potentially be recruited into the HrpL regulon via loss of transcription termination regulation and subsequent transcriptional read-through.

### Recruitment of a novel gene cluster into an *avrD*-containing virulence operon in *Pph*_1448A_

We identified a cluster of HrpL-regulated genes, *PSPPH_A0106-A0112*, downstream from *avrD* that were recruited into a novel HrpL-regulated operon transcribed from the *avrD* promoter. These genes are flanked in *Pla*_107_ and *Pph*_1448A_, by transposable elements, suggesting that they could be acquired by horizontal gene transfer (Figure 6). Deletion mutants of either *PSPPH_A0107* or *avrD* resulted in reduced virulence on French bean. The slightly reduced virulence we observed is in conflict with observations that allelic replacement of *avrD* by the *nptII* gene did not result in any growth defect in completive index assays [72]. This discrepancy could be explained by transcription from the *nptII* promoter in the previous work, or by the use of different growth assays, time points, and bean cultivars.

The *PSPPH_A0106-A0112* operon is most likely involved in small molecule(s) synthesis promoting bacterial growth on host plants. Component(s) synthesized by the products of this operon and their effect on plants remain to be determined. However, since syringolides can be made from AvrD-expressing *E. coli*, and since the *PSPPH_A0106-A0112* operon is not present in *E. coli*, we speculate that that the *PSPPH_A0106-A0112* operon is not required for syringolide production. When present, AvrD shares no less than 84% amino acid identity across *P. syringae* strains. Genes encoding an AvrD-like protein with about 30% identity are widely distributed among bacteria, including *Bacillus, Streptomyces* and *Vibrio*. In general, these *avrD*-like genes are not found as singletons, but instead are linked to genes encoding various enzymes not related to any of the *PSPPH_A0112-A0106* genes. In *Streptomyces coelicolor* A3(2), *AvrD* is part of an *mmy* operon responsible for synthesis of methylenomycin [88]. The *PSPPH_A0110* to *PSPPH_A0107* locus and to some extent the *PSPPH_A0106* genes have similarity to genes in operons from *Xanthomonas*, *Acidovorax*, *Pectobacterium* and *Ralstonia*. Only the *Ralstonia solanacearum* PSI07 megaplasmid, carries both an *avrD*-like gene and a *PSPPH_A0110-A0106* cluster of genes, but they are not contiguous on this plasmid. *PSPPH_A0112* is mainly limited to *P. syringae*, but shares some homology with HMPREF9336_00100 (29% amino acid identity) found in *Segniliparus rugosus* ATCC BAA-974, an opportunistic pathogen associated with mammalian lung disease [89]. *HMPREF9336_00100* and an *avrD*-like gene are linked in *Segniliparus rugosus*, being separated by only two genes and encoded on the same strand. We additionally observed that this operon has been disrupted by insertion of a transposable element in *P. syringae* CC1629, reminiscent of transposon disruptions of T3E genes commonly observed across the *P. syringae* phylogeny [3].

*hrpL* is widely distributed, and tightly linked in all *hrp/hrc* group I T3SS [1] and the non-canonical T3SS found in some *P. syringae*, as well as the T3SS of *P. viridiflava*, *P. fluorescens*, *Erwinia*, *Pantoea stewartii*, and *Dickeya*. It is the key virulence regulator in most if not all of these species. Our work highlights the advantages of integrating next generation transcriptional and genomic data to better understand the role of non-T3E HrpL regulon genes in plant-pathogen interactions. Our approach is readily applied to strains with sequenced genomes and broad phylogenetic sampling [63] to better understand *P. syringae* virulence mechanisms and their evolution.

## Materials and Methods

### Bacterial strains and growth conditions

For maintenance and transformation, *P. syringae* were grown in King's B media (KB) at 28°C. *E. coli* DH5α was grown in Luria-Bertani (LB) media at 37°C. Antibiotics were used at the following concentrations: 50 µg/ml rifampicin, 25 µg/ml kanamycin, 10 µg/ml tetracycline, and 25 µg/ml gentamycin, according to vector selection. Strains used or analyzed in this study are listed with their abbreviation in Table S8.

### Cloning and plasmids

Native *hrpL* from the various *P. syringae* were PCR amplified using LA-Taq (TaKaRa) and oligonucleotides listed in Table S9 containing *XbaI* and *Hind III* sites, then cloned into pTOPO-TA (Invitrogen). The pTOPO-TA::*hrpL* was sequenced, digested with *XbaI* and *HindIII*, and cloned into *NheI/HindIII*-digested pCF340 (Newman and Fuqua, 1999) and designated pBAD::*hrpL*.

*Por_curated__02784, 04644, 03530, 01245, 04371* and their respective upstream region containing the *hrp*-box were PCR amplified using *Pfx* (Invitrogen) and primers described in Table S9. Resulting PCR fragments were cloned into pENTR-D-TOPO (Invitrogen) and sequenced. *Por_curated__04644* was amplified similarly using primers containing attB1/attB2 sites and cloned into the pDONR 207 vector (Invitrogen). All resulting constructs were sub-cloned into either the gateway-compatible pJC532 vector containing the in-frame Δ79avrRpt2 sequence for translocation assays or the pJC531 vector containing an in-frame HA sequence [3] to check for protein expression. All vectors used in this study were transformed into *P. syringae* strains using tri-parental mating with an *E. coli* helper strain containing pRK2013.

### Preparation of samples for RNA-seq analysis

*Pseudomonas* strains containing pBAD::*hrpL*_native_ or pBAD::EV were grown overnight at 28°C, in KB media supplemented with tetracycline, then sub-cultured in fresh media at OD_600_ = 0.2, and grown until OD_600_ = 0.4–0.5. Bacteria were washed twice with 10 mM MgCl_2_ and resuspended in minimal medium [48] (MM is 50 mMKPO_4_ pH 5.7, 7.6 mM (NH4)_2_SO_4_, 1.7 mM MgCl_2_, 1.7 mM NaCl) containing 10 mM mannitol and supplemented with 1% glycerol and 0.1% peptone which suppresses *hrpL* induction. Bacteria were then inoculated in supplemented minimal media at OD_600_ = 0.2, and incubated shaking for 30 min at 28°C. Expression of *hrpL* was induced by addition of 200 mM L-arabinose. Aliquots of cell culture were taken 1, 3, 5 hours post-induction and treated with RNAprotect reagent (Qiagen). RNA isolation was performed by using the RNeasy minikit (Qiagen). Isolated total RNA was treated twice with TURBO DNase (Ambion). Total RNAs derived from each time point were pooled at a 1 to 3 ratio. 10 µg of pooled total RNA was depleted of 16S and 23S ribosomal RNA using RiboMinus (Invitrogen). cDNA were prepared from ∼1 µg of ribosomal depleted RNA, using random hexamer primers and Superscript II reverse transcriptase (Invitrogen). Second strand cDNA was prepared using DNA polymerase I and Ribonuclease H (Invitrogen). Double stranded cDNA was purified using Qiaquick spin columns (Qiagen) and eluted with EB buffer. Double stranded cDNA was sheared using a Covaris Disruptor. Library was prepared according to the manufacturer's protocol (Illumina). Sequencing of the library was performed according the manufacturer's protocol on either Illumina GAII including single-end, 36 cycles or Illumina HiSeq 2000 including single-end, 70 cycles.

### Sequence mapping and analysis

We analyzed our RNA reads using the GENE-counter pipeline. For the *Pto*_DC3000_, *Psy*_B728A_, and *Pph*_1448A_ datasets, we used the publically available genomes provided by NCBI, along with the transcriptome constructed by NCBI's gene prediction pipeline. For the *Por*, *Pjap* and *Pla_107_* dataset, we used in-house assembly for the genome and used JGI's Integrated Microbial Genomes – Expert Review gene prediction pipeline for the transcriptome. All ribosomal RNA genes were excluded from the transcriptome file for all datasets. Transcriptome sequences for each strain were blasted against their corresponding genome and GFF files were constructed from the Blast reports using an in-house script. We processed the RNA reads and aligned the reads using the default parameters of GENE-counter's CASHX read mapping algorithm. Reads mapping to multiple genomic locations were excluded. Annotated genes were included in the analysis only if at least one read in one sample matched that gene which can lead to highly duplicated genes not being considered. The false discovery rate cutoff for determining differential expression was set to 0.05. We made a small modification to GENE-counter's findDGE.pl script that allowed for random seeding during the sample depth normalization process. By repeating the normalization process 300 times we generated *B*-values to measure and control for normalization effects. The GenBank accession (http://www.ncbi.nlm.nih.gov/) and Gold ID (http://img.jgi.doe.gov/cgi-bin/w/main.cgi) of the genomes used in this study are CP000058-CP000060, Gi04410, CP000075, Gi07003, Gi03478, and AE016853-AE016855. RNA-seq data have been deposited in NCBI Gene Expression Omnibus and will be accessible through GEO Series accession number GSE46930 (http://www.ncbi.nlm.nih.gov/geo/).

### Manual curation of data set and designation of HrpL regulon

First, protein sequences of genes found up-regulated in our analysis with *B*-values≥50% were used to search each genome used in this study with BlastP to identify genes split up in different contigs/scaffolds. Possible duplication was ruled out by comparing the size of the query to the size of the subject sequence (of complete genomes, principally). Putative sequencing errors leading to stop codons and discontinuous ORFs, led to consecutive queries matching the same subject sequence. Only the entry with the most significant *q*-value was kept. Secondly, genes encoding open reading frames shorter than 60 amino acids were excluded from our data set. Thirdly, loci of genes not previously found HrpL-dependent were assessed for linkage to genes with a *hrp*-box. As previously described [41], [90], we observed potential transcriptional read-through artifacts for which directly HrpL-targeted genes led to apparent up-regulation of adjacent genes. Therefore, genes found differentially expressed adjacent to a HrpL-regulated gene, but on the opposite DNA strand were considered to be putative transcriptional read-through and removed from our analysis. Genes encoded on the same strand as the HrpL-regulated gene were kept. Fourth, genes with a *hrp*-box embedded within their ORF on either sense or anti-sense strands were not included. Adjacent genes encoded on the same strand as the manually predicted *hrp*-box were included in the defined HrpL regulon, but genes on the opposite strand of the *hrp*-box were excluded. All genes removed from the HrpL regulons after manual curation are listed Table S4.

### Quantitative RT-PCR

For native gene expression, bacteria were grown for 4 hours in KB media from OD_600_ = 0.2, washed twice with sterile 10 mM MgCl_2_ and transferred into MM minimum media containing 10 mM mannitol for *Pto_DC3000_, Psy_B728a_, Pla_107_, Pja, Por* strains or MM minimum media containing 10 mM fructose for *Pph*_1448A_ strain. Cells were collected after 5 hours of incubation shaking at 28°C and treated with RNAprotect reagent (Qiagen). Total RNA derived from cells grown in MM media or arabinose inducing media (as above) was extracted using the RNeasy minikit (Qiagen), DNase treated twice (Ambion Turbo DNase), and cleaned up with Qiagen RNeasy Mini kit. Reverse transcription was performed using SuperScript II (Invitrogen) with 2 µg total RNA. Diluted cDNA was analyzed with SYBR green (Applied Biosystem) using the Opticon 2 System (BioRad). Primers used are listed in Table S9.

### Δ79*avrRpt2*-based translocation assay

Four week old Col-0 and Col-0 *rps2–101c* (*rps2*) plants were hand inoculated with *Pto*_DC300028E_ [57] carrying Δ79*avrRpt2* fusion clones at OD_600_ = 0.1. Plants were scored for Hypersensitive Response (HR) and pictures were taken 24 h after inoculation.

### Generation of *P. syringae* knockout mutants

Knockout constructs were generated using MTN1907, a modified version of pLVC-D which allows for SacB counter-selection [3], [91]. To create *Pph*_1448A_Δ*PSPPH_A0107, Pph_1448A_ΔPSPPH_A0113* mutants, 5′ and 3′ regions flanking the gene of interest were amplified using *Pfx* (Invitrogen) and combined by overlap extension PCR (Table S9), then cloned into pENTR-D-TOPO and sequenced. To generate the *Pph*_1448A_Δ*PSPPH_1855, Psy_B728a_*Δ*hrpL* and *PorΔhrpL* mutants, nucleotide sequences corresponding to the fused flanking regions of each gene were synthesized including Gateway recombination sites and cloned in the pUC17 vector (GenScript). All five clones were recombined into MTN1907 and transformed into either *Pph_1448A_, Psy_B728a_* or *Por* by tri-parental mating. After selection on tetracycline plates, merodiploids resulting from homologous recombination were verified by PCR. Two independent merodiploids carrying either a 3′ or 5′ insertion were grown on KB agar containing 5% sucrose to select for the loss of *sacB* via a second recombination event. Putative clean-deletion mutants were verified by PCR using flanking primers and gene specific primers.

### Bacterial growth on French bean

Before inoculation, *Pph*_1448A_ and mutants were grown overnight and sub-cultured from OD_600_ = 0.2 for 4 hours in KB media, then washed twice with 10 mM MgCl_2_. Two week old French bean cv. Tendergreen improved (Livingston Seed Co.) were dip inoculated with freshly grown bacteria at OD_600_ = 0.001 bacteria in 10 mM MgCl_2_ and 0.04% Silwet L-77. Four plants were dip inoculated for each strain. Three days and an half after inoculation leaf discs were cored (12 to 16 replicates, each 4 cores), ground in 10 mM MgCl_2_, serially diluted and plated on KB/50 µg/ml rifampicin and bacteria counted. Each set of mutants were tested side by side with the wild type strain at least 3 times.

## Supporting Information

Figure S1 Graphical representation of our experimental pipeline. Isogenic *P. syringae* strains carrying either pBAD::EV or pBAD::*hrpL* were grown on MM media supplemented with arabinose and collected 1, 3 and 5 hours post induction. RNA was extracted for each time point and cDNA prepared to confirm induction of *hrpL* and *hrcC* for *P. syringae* pBAD::*hrpL*. Total RNA for each time point was pooled equally. Pooled RNA for each strain was subjected to rRNA removal and double stranded cDNA prepared (Materials and Methods). Illumina libraries were prepared according to manufacturer's protocol and sequenced. Resulting reads were used to run GENE-counter. After 300 bootstraps of GENE-counter, genes from each sample were assigned a median read count, a median *p* and *q*-value as well as a *B*-value.
(TIF)

Figure S2 Detailed results of qRT-PCRs described in Table 3 from samples derived from the pBAD system. (**A**) For *Pto*_DC3000_ genes, (**B**) for *Pph*_1448A_ genes, (**C**) for *Psy*_B728a_ genes, (**D**) for *Por* genes. cDNA was prepared from the same total RNA used to generate our RNA-seq data for all strains except *Pto*_DC3000_. For *Pto*_DC3000_, cDNAs were prepared from an independent biological replicate. Expression was normalized to *gap-1*. For determination of the relative expression, each EV sample was set to 1 and HrpL samples normalized to the corresponding EV samples. Error bars represent SD.
(TIF)

Figure S3 Detailed results of qRT-PCRs described in Table 3 from samples derived from isogenic strains grown in MM media. (**A**) For *Pto*_DC3000_ genes, (**B**) for *Pph*_1448A_ genes, (**C**) for *Psy*_B728a_ genes, (**D**) for *Por* genes. cDNA was prepared from wild type strains and corresponding isogenic Δ*hrpL* mutants grown in MM media for 5 hours. Expression was normalized to *gap-1*. For determination of the relative % expression, each wild type strain sample was set to 100% and Δ*hrpL* samples normalized accordingly. Error bars represent SD.
(TIF)

Figure S4 The majority of effector genes are found up-regulated in our analysis. Effector suites present in each strain are listed. In bold, effector genes found up-regulated by RNA-seq. Underlined, effector genes HrpL-dependent according to *Pseudomonas syringae* Genome Resources, (PPI database http://www.pseudomonas-syringae.org/) but not found up-regulated in our analysis. In grey, effector genes previously identified according to a combination of homology and functional criteria described in Chang et al., 2005 and Baltrus et al., 2011 but not found to be HrpL-regulated in these strains in any experiment, to our knowledge. ‘ indicates insertion or truncation according to PPI database. The new *Por* type III effectors defined in this study are listed in red.
(TIF)

Figure S5 *hopBH1* and *hopBI1* are both present across phylogenetically diverse strains *of P. syringae*. (**A**) Bayesian phylogenetic tree of 45 *Pseudomonas* strains [3], [40], [63] based on seven conserved loci as described in [3]. Bayesian posterior probabilities are displayed on the phylogeny only at nodes where these values are <0.95. Each phylogenetic group (defined according to Berge et al., personal communication and [27]) is color coded. (**B**) Distribution of *hopBH1* and *hopBI1* across the 45 *Pseudomonas* strains. Dark blue boxes indicate presence of corresponding full length ORF. Light Blue box indicates truncated ORF. White boxes indicate absence of corresponding ORF in the sequenced genome.
(TIF)

Figure S6 Amino-acid sequence alignment of HopBH1. Alignment performed using clustal W with sequences from all *P. syringae* strains known to date to contain *hopBH1*.
(TIF)

Figure S7 Expression of *PSPTO_2105* and *2130* or their orthologs under native conditions supports our results obtained with arabinose-inducible *hrpL* system. (**A**) Relative expression of *PSPTO_2130* and its orthologs. (**B**) Relative expression of *PSPTO_2105* and its orthologs. qRT-PCR analysis was performed on RNA samples derived from wild type strains and the cognate isogenic Δ*hrpL* mutant grown in MM media. Expression was normalized to *gap-1*. For determination of the relative expression, each EV sample was set to 1 and HrpL samples normalized to the corresponding EV samples. Error bars represent SD.
(TIF)

Figure S8 HrpL-dependent up-regulation of genes downstream of *PSPTO_2130* and its orthologs in *Pto*_DC3000_, *Pph*_1448A_, but not *Psy*_B728a_. (**A**), Graphical representation of *PSPTO_2131-PSPTO-2128* operon. qRT-PCR analysis was performed on RNA samples derived from (**B**) *Pto*_DC3000_(pBAD::*hrpL*) and *Pto*_DC3000_(pBAD::EV) (**C**) *Pph*_1448A_(pBAD::*hrpL*) and *Pph*_1448A_(pBAD::EV); and (**D**) *Psy*_B728a_(pBAD::*hrpL*) and *Psy*_B728a_(pBAD::EV). ORF nomenclature for operons from *Pph*_1448A_ and *Psy*_B728a_ in **C** and **D**, respectively, is listed directly under the corresponding ORF numbers in *Pto*_DC3000_ in **B**. Expression was normalized to *gap-1*. For determination of the relative expression, each EV sample was set to 1 and HrpL samples normalized to the corresponding EV samples. Error bars represent SD. qRT-PCR analysis was performed on RNA samples derived from (**E**) *Pto*_DC3000_ and *Pto*_DC3000_ *ΔhrpL* (**F**) *Pph*_1448A_ and *Pph*_1448A_ *ΔhrpL*, For determination of the relative % expression, each wild type strain sample was set to 100% and Δ*hrpL* samples normalized accordingly. Error bars represent SD.
(TIF)

Figure S9 *Pph*_1448A_ mutants deleted in thiamine biosynthesis lipoprotein gene display reduced growth on Tendergreen beans. Two week old bean cv. Tendergreen beans were dip inoculated with wild type *Pph*_1448A_ *(Pph)* or two independent mutants with a clean deletion of *PSPPH_1855* (*Pph*Δ1855 #1, *Pph*Δ1855 #2), at OD_600_ = 0.001. Bacterial growth of each strain was determined after 3.5 dpi. Error bars represent SD. This experiment was repeated twice with similar results.
(TIF)

Figure S10 Determination of the relative expression of *avrD PSPPH_A0112, _A0107, _A0106* in various *Pph*_1448A_ mutants. qRT-PCR was performed on cDNA derived from wild type *Pph*_1448A_ *(Pph)*, *Pph*_1448A_Δ*hrpL*, two independent clean deletion *avrD* mutants (Δa*vrD* #1, Δa*vrD* #2), and two independent clean deletion *PSPPH_A0107* mutants (Δ*A0107* #1, Δ*A0107* #2). Expression was normalized to *gap-1*. For determination of the relative expression, each EV sample was set to 1 and HrpL samples normalized to the corresponding EV samples. Error bars represent SD. This experiment was repeated 3 times with similar results.
(TIF)

Table S1 Raw bootstrapped GENE-counter results for each strain.
(XLSX)

Table S2 Genes found down-regulated by RNA-seq.
(XLSX)

Table S3 Our defined HrpL regulons across various *P. syringae* after manual curation.
(XLSX)

Table S4 List of genes excluded from analysis after manual curation.
(XLSX)

Table S5 List of “confirmed” HrpL-regulated genes for *Pto*_DC3000_ and *Pph*_1448A_ according to previous studies.
(XLSX)

Table S6 BlastP search results of the product of each HrpL regulon gene across the strains tested in this study.
(XLSX)

Table S7 Details on the attribution of the functional classification for each HrpL regulon gene.
(XLSX)

Table S8 List of *Pseudomonas* strains used or analyzed in this study.
(XLSX)

Table S9 List of primers and sequences used in this study.
(XLSX)
