# Supplementary figures and images for: Variable Suites of Non-effector Genes Are Co-regulated in the Type III Secretion Virulence Regulon across the Pseudomonas syringae Phylogeny
Source: PLoS Pathog. 2014 Jan 2;10(1):e1003807. doi: 10.1371/journal.ppat.1003807 (PMC3879358; doi:10.1371/journal.ppat.1003807)

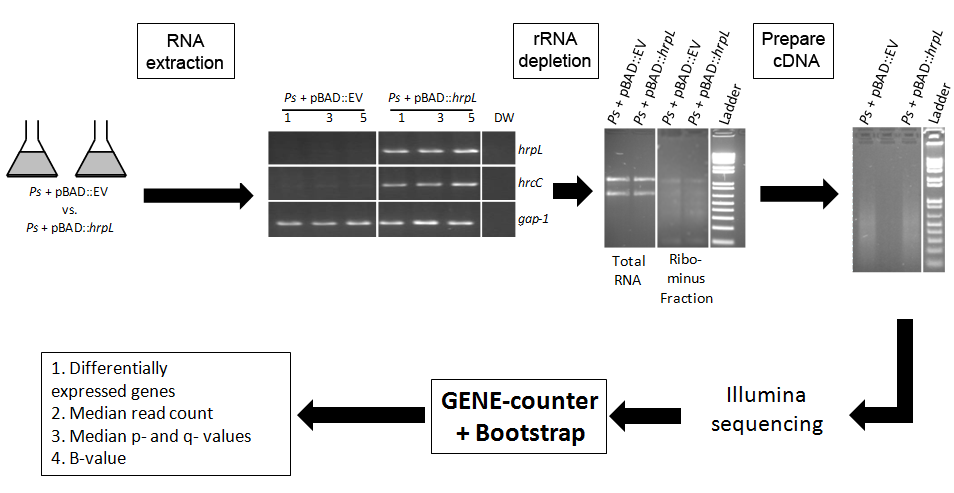

Supplement: Figure S1 — Graphical representation of our experimental pipeline. Isogenic P. syringae strains carrying either pBAD::EV or pBAD::hrpL were grown on MM media supplemented with arabinose and collected 1, 3 and 5 hours post induction. RNA was extracted for each time point and cDNA prepared to confirm induction of hrpL and hrcC for P. syringae pBAD::hrpL. Total RNA for each time point was pooled equally. Pooled RNA for each strain was subjected to rRNA removal and double stranded cDNA prepared (Materials and Methods). Illumina libraries were prepared according to manufacturer's protocol and sequenced. Resulting reads were used to run GENE-counter. After 300 bootstraps of GENE-counter, genes from each sample were assigned a median read count, a median p and q-value as well as a B-value. (TIF) [file ppat.1003807.s001.tif]

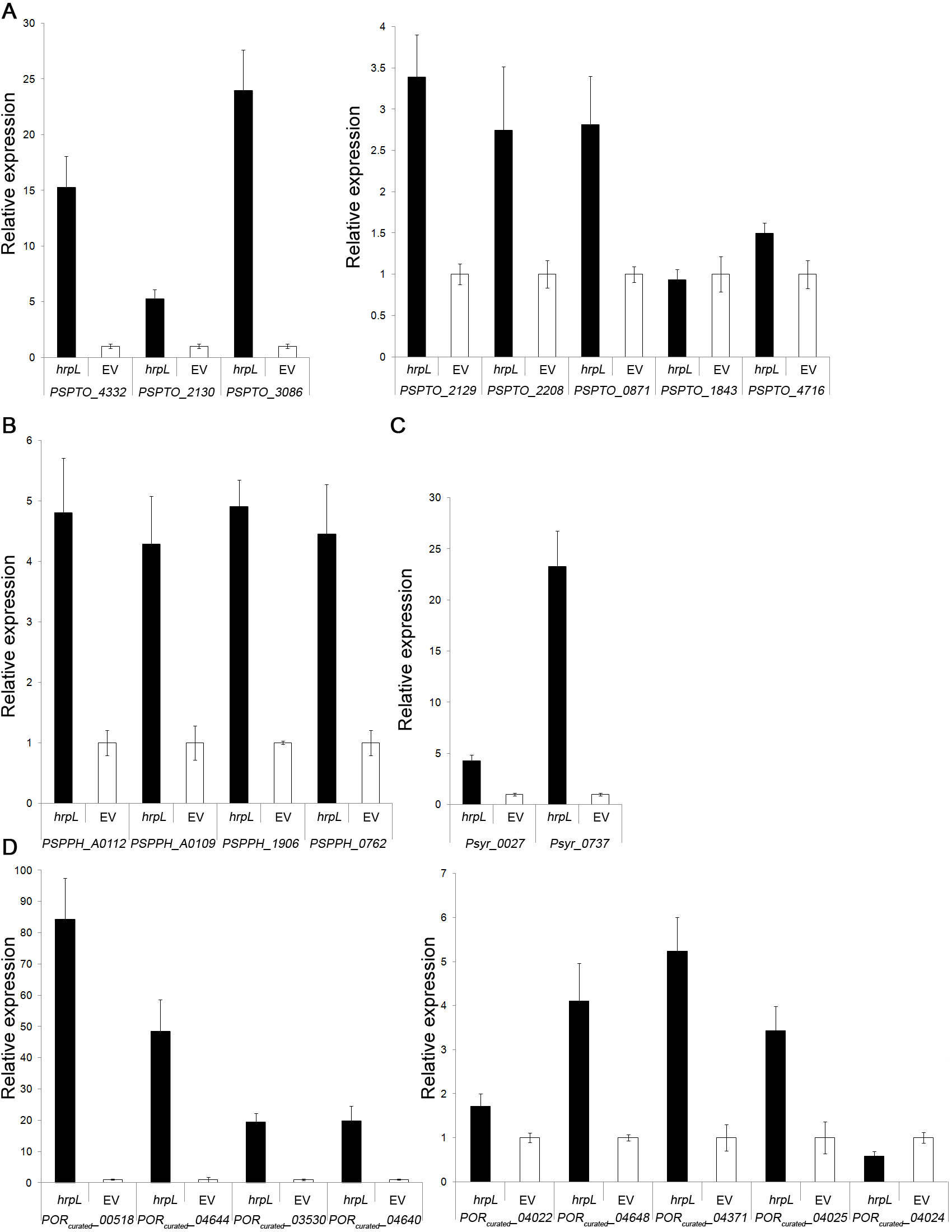

Supplement: Figure S2 — Detailed results of qRT-PCRs described in Table 3 from samples derived from the pBAD system. (A) For Pto DC3000 genes, (B) for Pph 1448A genes, (C) for Psy B728a genes, (D) for Por genes. cDNA was prepared from the same total RNA used to generate our RNA-seq data for all strains except Pto DC3000. For Pto DC3000, cDNAs were prepared from an independent biological replicate. Expression was normalized to gap-1. For determination of the relative expression, each EV sample was set to 1 and HrpL samples normalized to the corresponding EV samples. Error bars represent SD. (TIF) [file ppat.1003807.s002.tif]

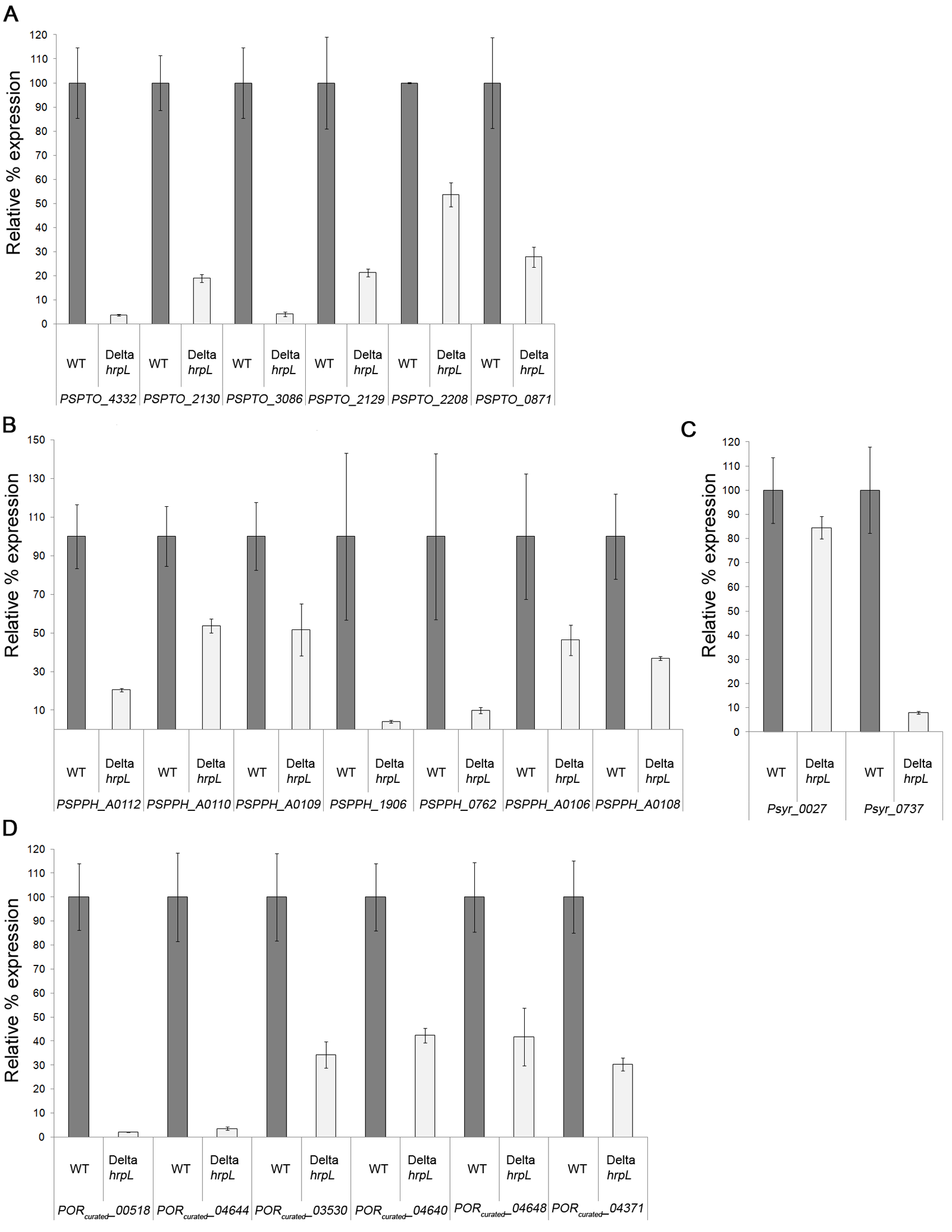

Supplement: Figure S3 — Detailed results of qRT-PCRs described in Table 3 from samples derived from isogenic strains grown in MM media. (A) For Pto DC3000 genes, (B) for Pph 1448A genes, (C) for Psy B728a genes, (D) for Por genes. cDNA was prepared from wild type strains and corresponding isogenic ΔhrpL mutants grown in MM media for 5 hours. Expression was normalized to gap-1. For determination of the relative % expression, each wild type strain sample was set to 100% and ΔhrpL samples normalized accordingly. Error bars represent SD. (TIF) [file ppat.1003807.s003.tif]

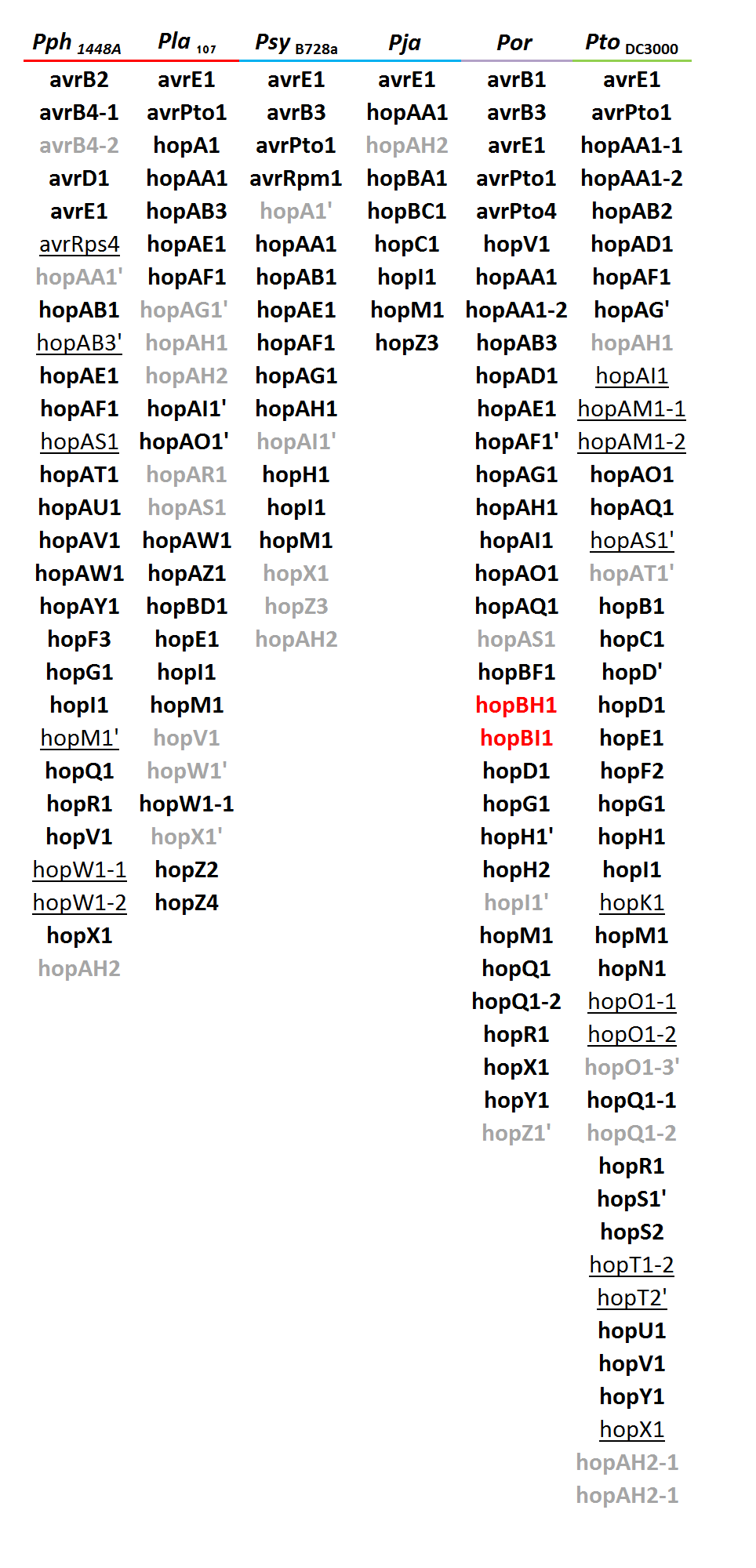

Supplement: Figure S4 — The majority of effector genes are found up-regulated in our analysis. Effector suites present in each strain are listed. In bold, effector genes found up-regulated by RNA-seq. Underlined, effector genes HrpL-dependent according to Pseudomonas syringae Genome Resources, (PPI database http://www.pseudomonas-syringae.org/) but not found up-regulated in our analysis. In grey, effector genes previously identified according to a combination of homology and functional criteria described in Chang et al., 2005 and Baltrus et al., 2011 but not found to be HrpL-regulated in these strains in any experiment, to our knowledge. ‘ indicates insertion or truncation according to PPI database. The new Por type III effectors defined in this study are listed in red. (TIF) [file ppat.1003807.s004.tif]

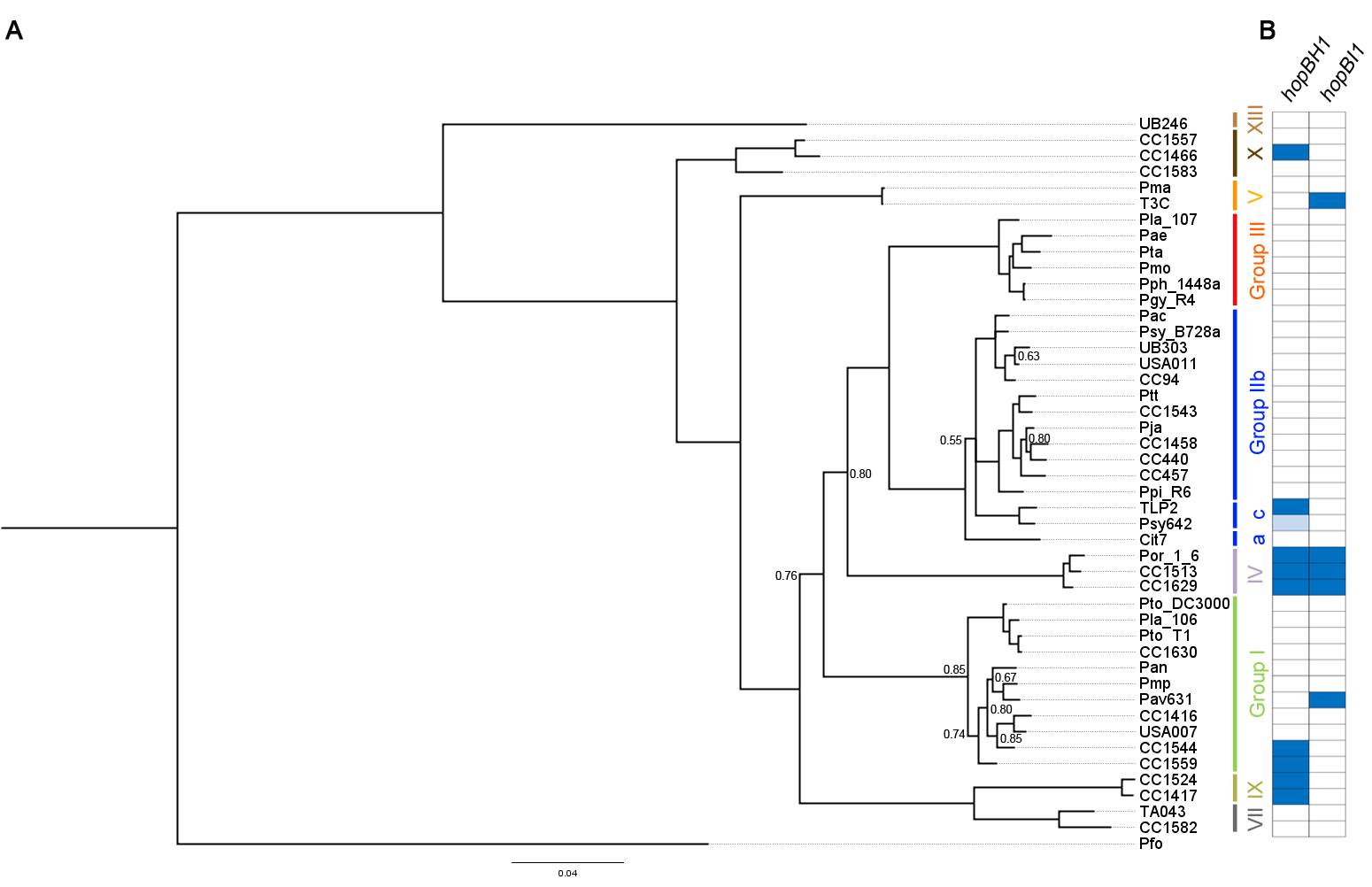

Supplement: Figure S5 — hopBH1 and hopBI1 are both present across phylogenetically diverse strains of P. syringae. (A) Bayesian phylogenetic tree of 45 Pseudomonas strains [3], [40], [63] based on seven conserved loci as described in [3]. Bayesian posterior probabilities are displayed on the phylogeny only at nodes where these values are <0.95. Each phylogenetic group (defined according to Berge et al., personal communication and [27]) is color coded. (B) Distribution of hopBH1 and hopBI1 across the 45 Pseudomonas strains. Dark blue boxes indicate presence of corresponding full length ORF. Light Blue box indicates truncated ORF. White boxes indicate absence of corresponding ORF in the sequenced genome. (TIF) [file ppat.1003807.s005.tif]

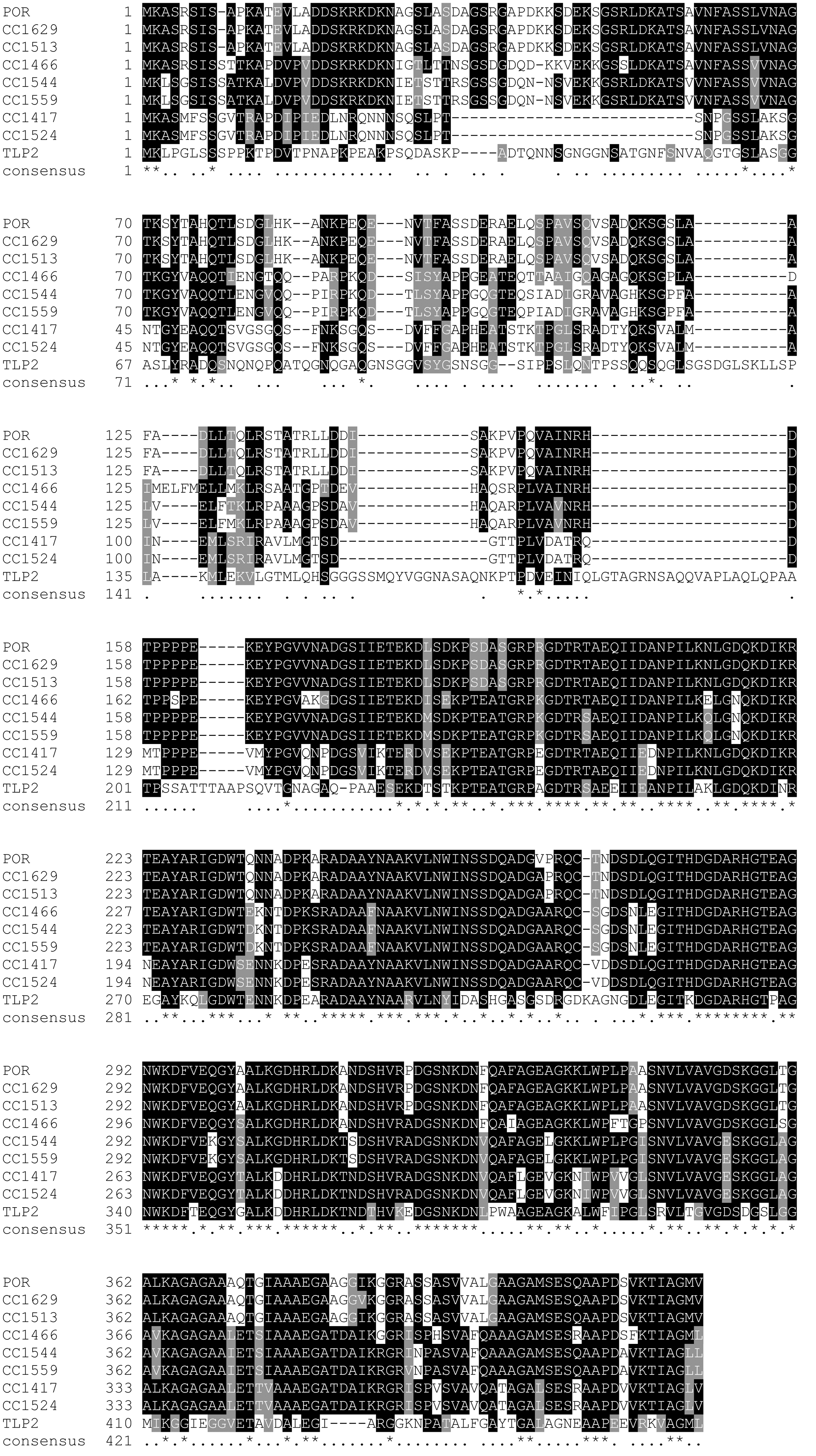

Supplement: Figure S6 — Amino-acid sequence alignment of HopBH1. Alignment performed using clustal W with sequences from all P. syringae strains known to date to contain hopBH1. (TIF) [file ppat.1003807.s006.tif]

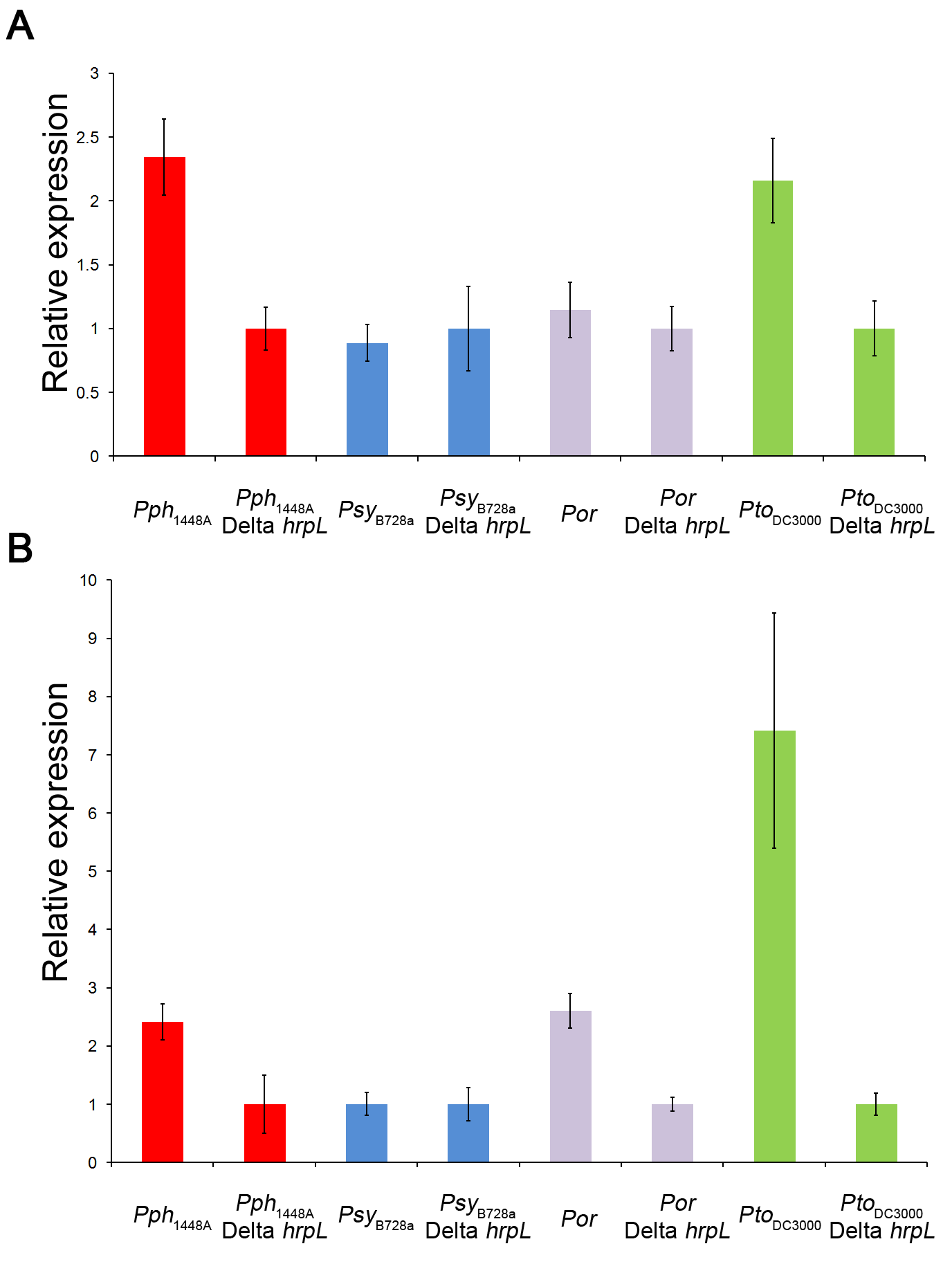

Supplement: Figure S7 — Expression of PSPTO_2105 and 2130 or their orthologs under native conditions supports our results obtained with arabinose-inducible hrpL system. (A) Relative expression of PSPTO_2130 and its orthologs. (B) Relative expression of PSPTO_2105 and its orthologs. qRT-PCR analysis was performed on RNA samples derived from wild type strains and the cognate isogenic ΔhrpL mutant grown in MM media. Expression was normalized to gap-1. For determination of the relative expression, each EV sample was set to 1 and HrpL samples normalized to the corresponding EV samples. Error bars represent SD. (TIF) [file ppat.1003807.s007.tif]

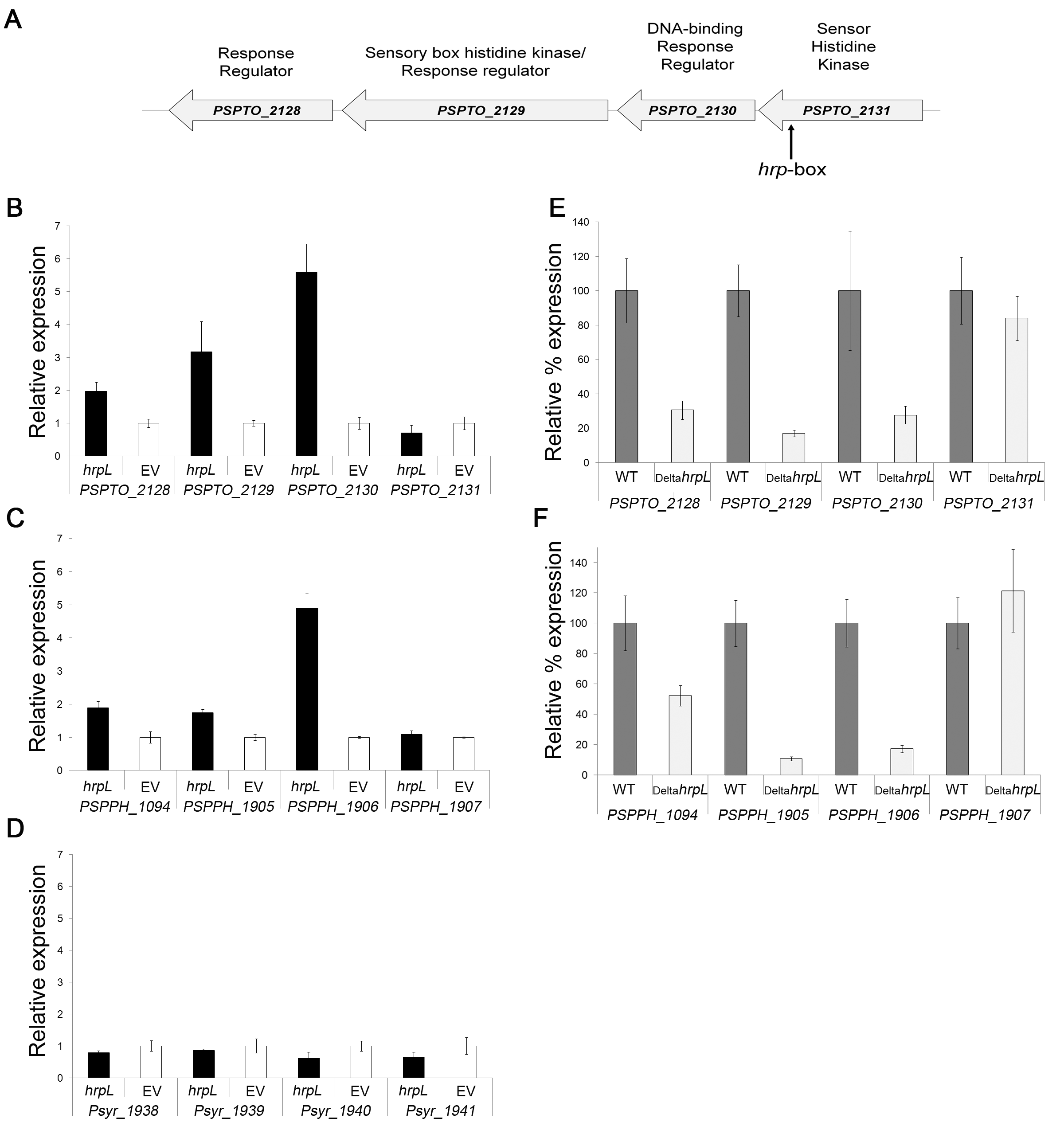

Supplement: Figure S8 — HrpL-dependent up-regulation of genes downstream of PSPTO_2130 and its orthologs in Pto DC3000, Pph 1448A, but not Psy B728a. (A), Graphical representation of PSPTO_2131-PSPTO-2128 operon. qRT-PCR analysis was performed on RNA samples derived from (B) Pto DC3000(pBAD::hrpL) and Pto DC3000(pBAD::EV) (C) Pph 1448A(pBAD::hrpL) and Pph 1448A(pBAD::EV); and (D) Psy B728a(pBAD::hrpL) and Psy B728a(pBAD::EV). ORF nomenclature for operons from Pph 1448A and Psy B728a in C and D, respectively, is listed directly under the corresponding ORF numbers in Pto DC3000 in B. Expression was normalized to gap-1. For determination of the relative expression, each EV sample was set to 1 and HrpL samples normalized to the corresponding EV samples. Error bars represent SD. qRT-PCR analysis was performed on RNA samples derived from (E) Pto DC3000 and Pto DC3000 ΔhrpL (F) Pph 1448A and Pph 1448A ΔhrpL, For determination of the relative % expression, each wild type strain sample was set to 100% and ΔhrpL samples normalized accordingly. Error bars represent SD. (TIF) [file ppat.1003807.s008.tif]

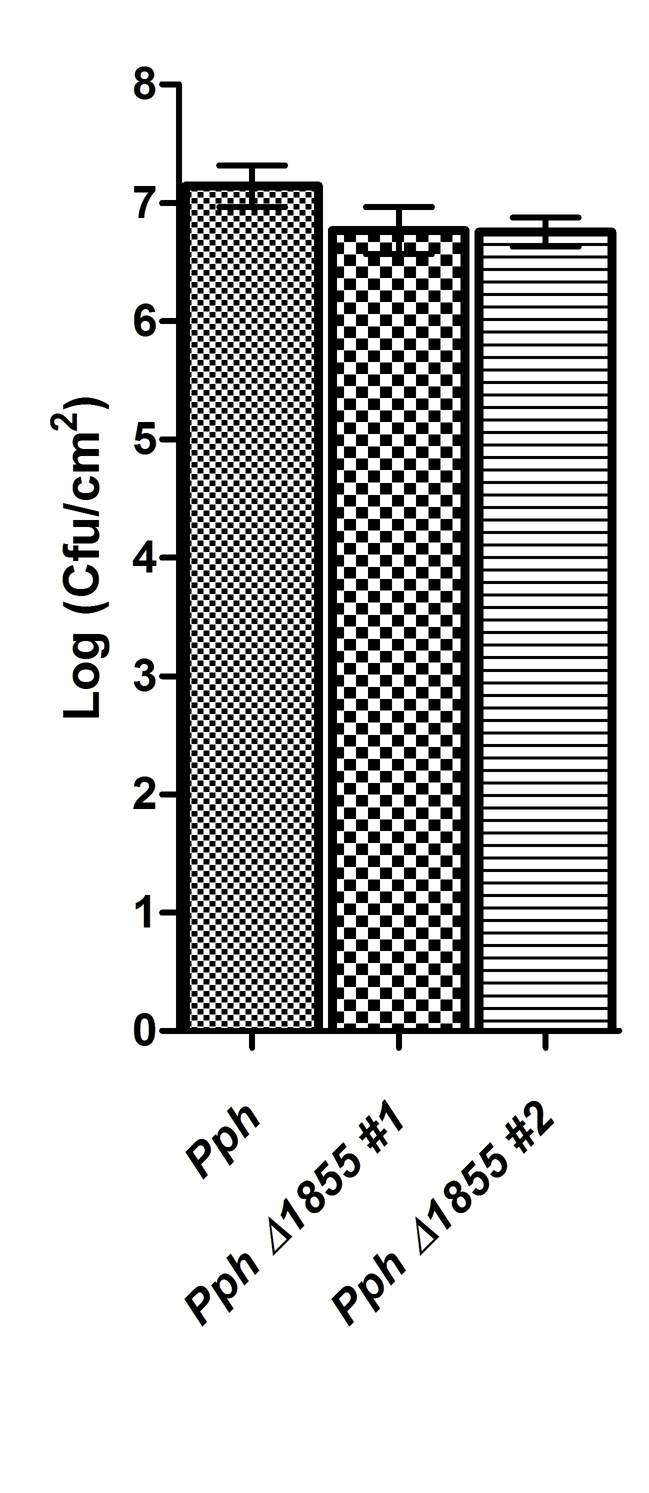

Supplement: Figure S9 — Pph 1448A mutants deleted in thiamine biosynthesis lipoprotein gene display reduced growth on Tendergreen beans. Two week old bean cv. Tendergreen beans were dip inoculated with wild type Pph 1448A (Pph) or two independent mutants with a clean deletion of PSPPH_1855 (PphΔ1855 #1, PphΔ1855 #2), at OD600 = 0.001. Bacterial growth of each strain was determined after 3.5 dpi. Error bars represent SD. This experiment was repeated twice with similar results. (TIF) [file ppat.1003807.s009.tif]

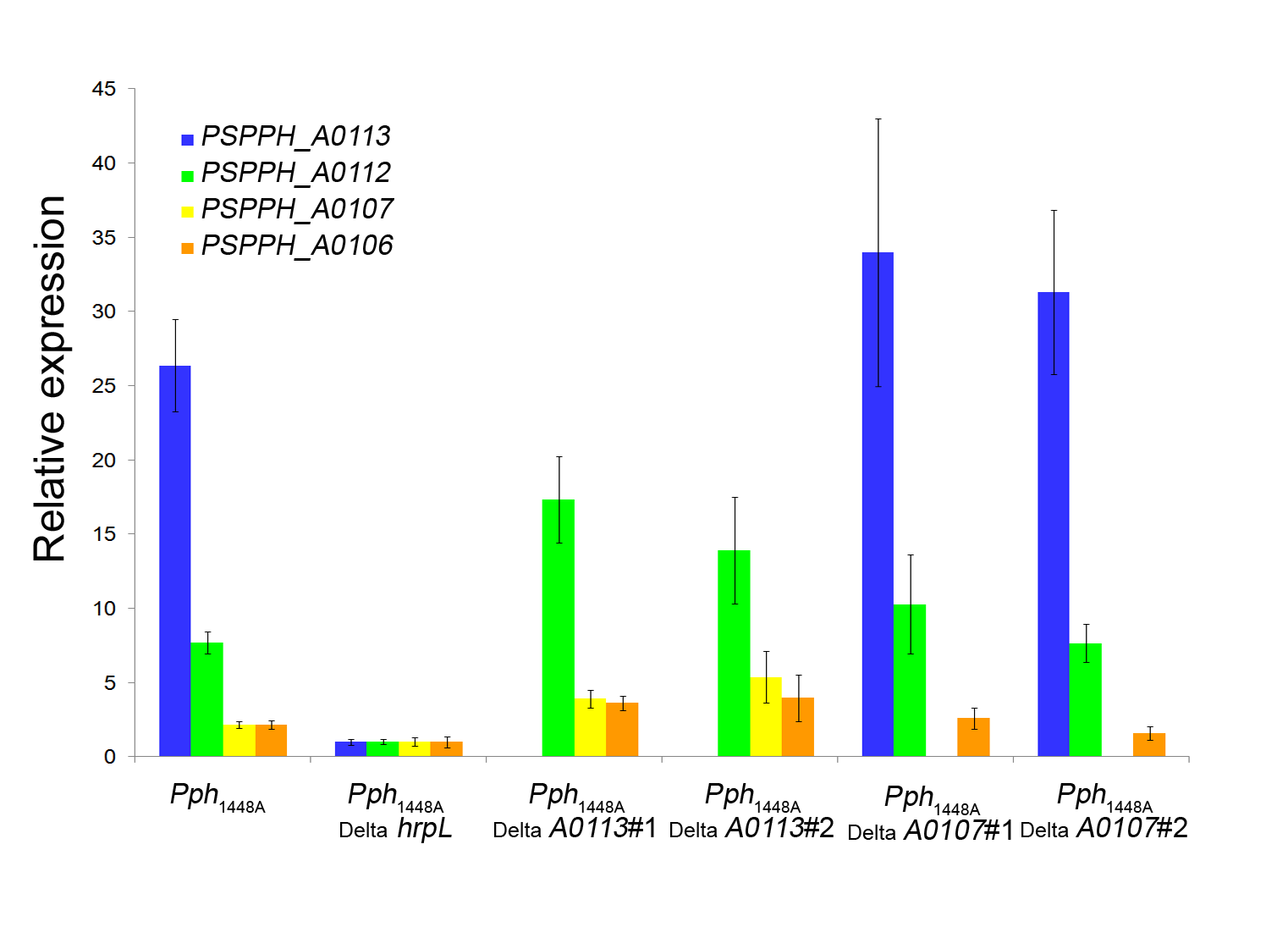

Supplement: Figure S10 — Determination of the relative expression of avrD PSPPH_A0112, _A0107, _A0106 in various Pph 1448A mutants. qRT-PCR was performed on cDNA derived from wild type Pph 1448A (Pph), Pph 1448AΔhrpL, two independent clean deletion avrD mutants (ΔavrD #1, ΔavrD #2), and two independent clean deletion PSPPH_A0107 mutants (ΔA0107 #1, ΔA0107 #2). Expression was normalized to gap-1. For determination of the relative expression, each EV sample was set to 1 and HrpL samples normalized to the corresponding EV samples. Error bars represent SD. This experiment was repeated 3 times with similar results. (TIF) [file ppat.1003807.s010.tif]
